# Supplementary material for: Seroprevalence of anti-AAV neutralizing antibodiesin healthy and retinitis pigmentosa cohorts: A multi-province study in China
Source: Mol Ther Adv. 2026 Apr 28;34(2):201743. doi: 10.1016/j.omta.2026.201743 (PMC13254674; doi:10.1016/j.omta.2026.201743)
Supplement: Document S1. Figures S1–S3 and Tables S1–S9 [file mmc1.pdf]

OMTA, Volume *34*

## Supplemental information

# Seroprevalence of anti-AAV neutralizing antibodies in healthy and retinitis pigmentosa cohorts: A multi-province study in China

**Jingxiao Du, Huixun Jia, Xuewei Xie, Xiaosa Li, Ting Zhang, Yidong Wu, Zishi Liu, Haoliang Zhang, Zhiyan Li, Weihua Gu, Xinkun Tao, Jieqiong Chen, Junran Sun, Tong Li, and Xiaodong Sun**

## SUPPLEMENTAL MATERIAL

### Supplemental Figures

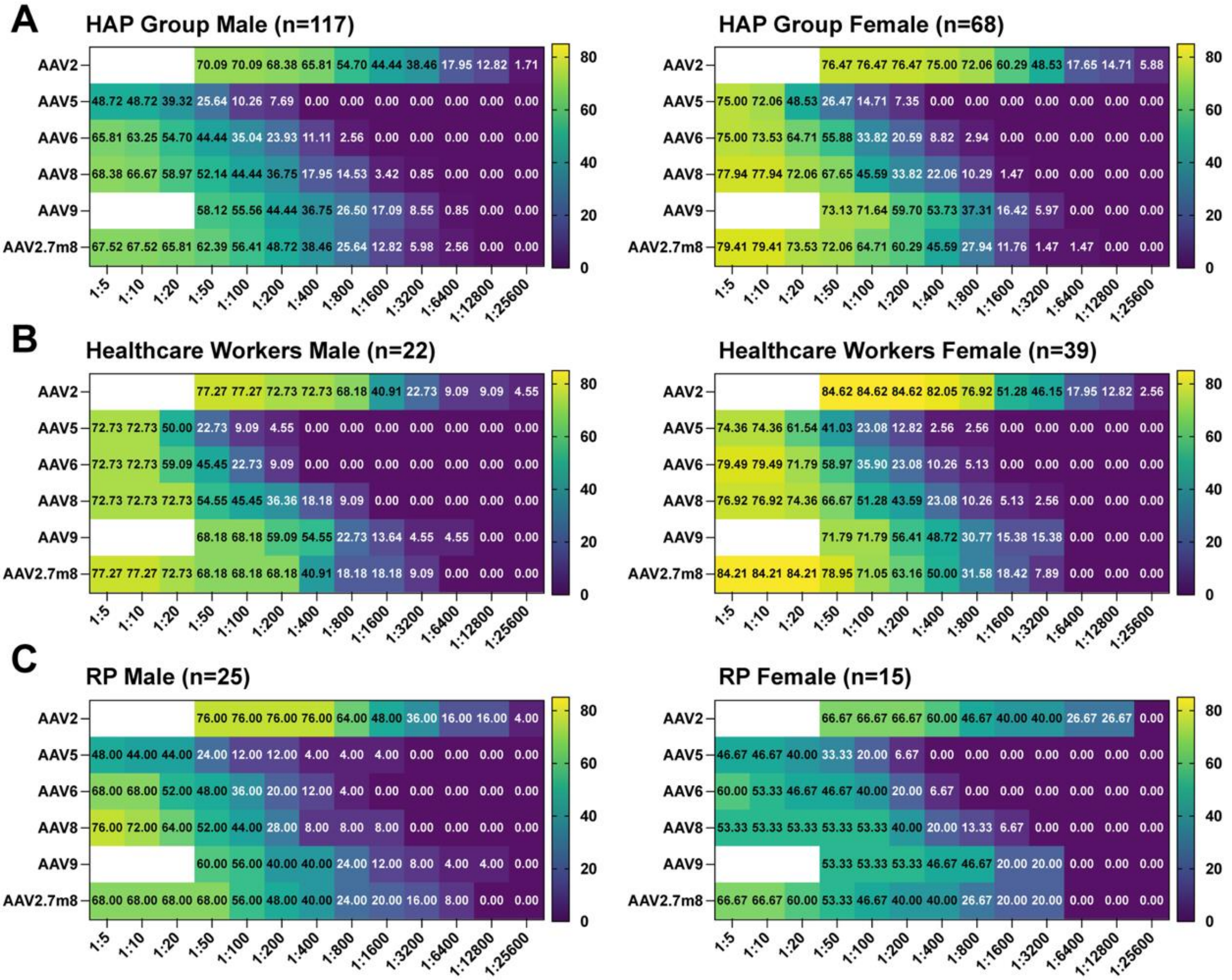


**Figure S1. Gender-specific NAbs seroprevalence across different dilution gradients in the HAP, healthcare workers, and RP groups.**

(A) Heatmap of anti-AAV NAbs seroprevalence by sex in HAP group for AAV2, AAV5, AAV6, AAV8, AAV9, and AAV2.7m8.

(B) Heatmap of anti-AAV NAbs seroprevalence by sex in healthcare workers for AAV2, AAV5, AAV6, AAV8, AAV9, and AAV2.7m8.

(C) Heatmap of anti-AAV NAbs seroprevalence by sex in RP group for AAV2, AAV5, AAV6, AAV8, AAV9, and AAV2.7m8.

The percentage of seropositive individuals is indicated by color intensity according to the scale, with numbers inside squares representing the exact proportion at each serotype and dilution level.

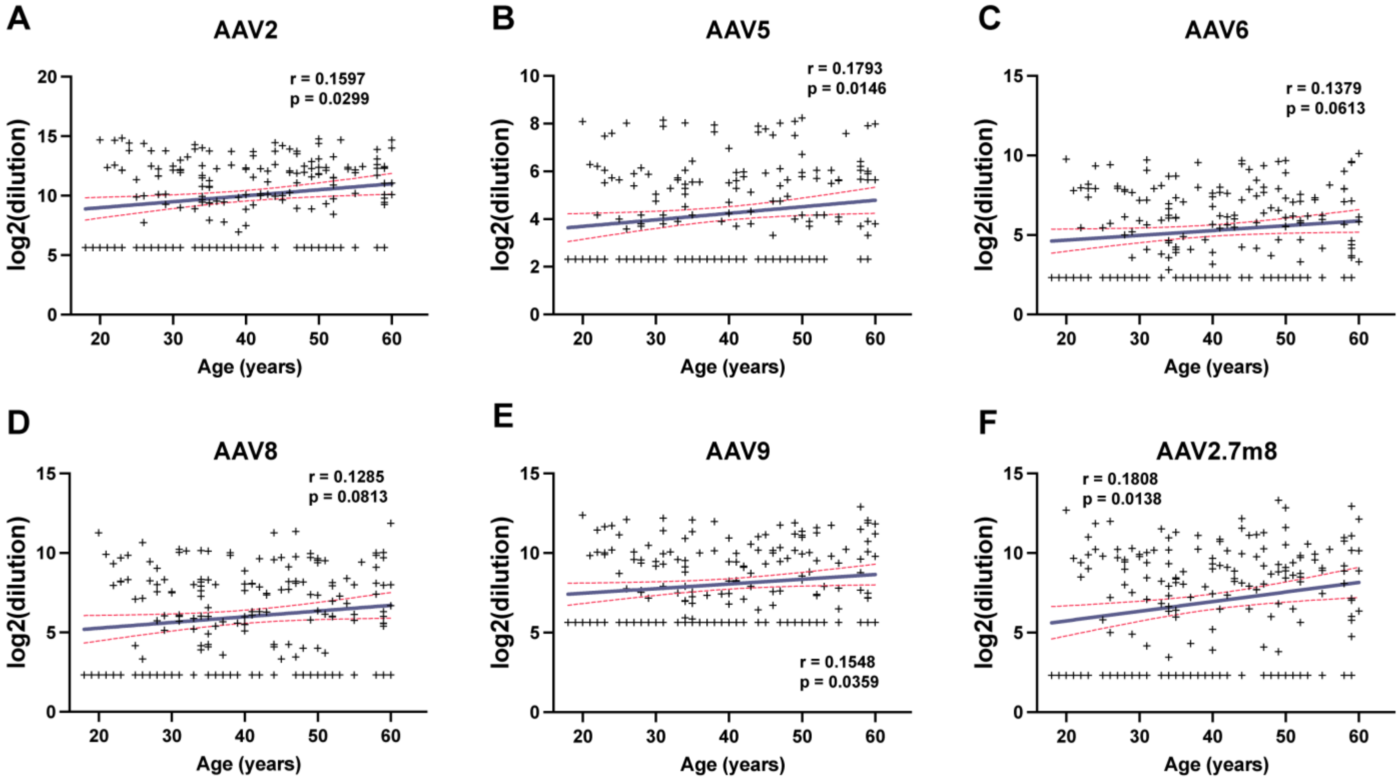


**Figure S2. Correlations between anti-AAV NAb titers and age in the HAP group.**

(A–F) Scatter plots illustrating the correlations between NAb titers against AAV2, AAV5, AAV6, AAV8, AAV9, and AAV2.7m8 and participant age. Blue solid line: fitted simple linear regression. Red dashed line: 95% confidence interval for the fitted regression. r, Spearman’s correlation coefficient. Correlations were calculated using log2-transformed NAb titers (ID50).

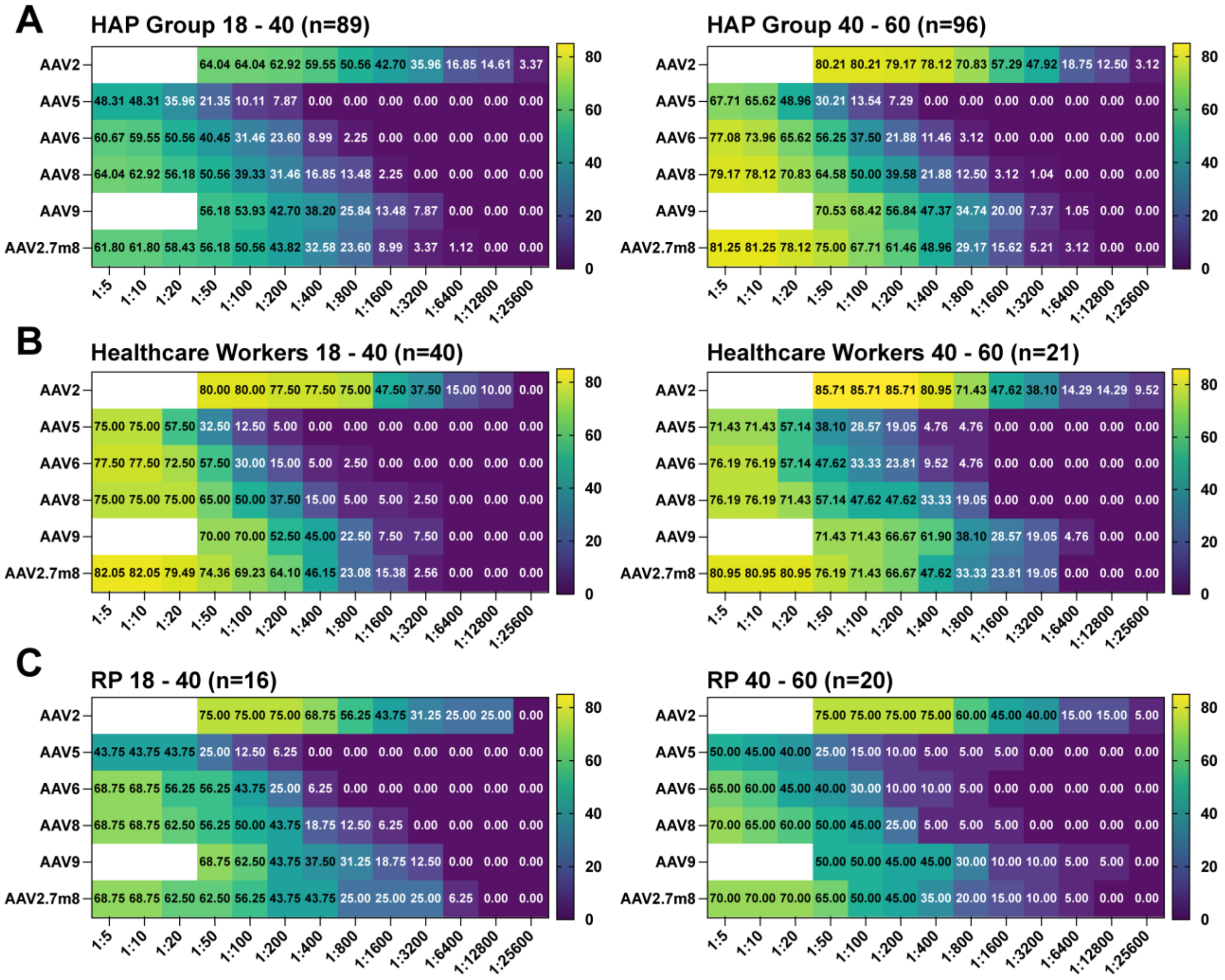


**Figure S3. Age-specific anti-AAV NAb seroprevalence across dilution gradients in the HAP, healthcare worker, and RP groups.**

(A) Heatmap of age-specific anti-AAV NAb seroprevalence in the HAP group for AAV2, AAV5, AAV6, AAV8, AAV9, and AAV2.7m8.

(B) Heatmap of age-specific anti-AAV NAb seroprevalence in healthcare workers for AAV2, AAV5, AAV6, AAV8, AAV9, and AAV2.7m8.

(C) Heatmap of age-specific anti-AAV NAb seroprevalence in the RP group for AAV2, AAV5, AAV6, AAV8, AAV9, and AAV2.7m8.

The percentage of seropositive individuals is indicated by color intensity according to the scale, with numbers inside squares representing the exact proportion at each serotype and dilution level.

**Supplemental Tables**

**Table S1. NAbs characteristics by gender in adult controls, healthcare workers and RP patients**

| Group | Serotype | Gender | Median | 95% CI | Minimum | Maximum | p-value |
|---|---|---|---|---|---|---|---|
| HAP | AAV2 | Male | 1:998 | 1:665 - 1:2903 | 1:50 | 1:26578 | 0.152 |
| | | Female | 1:2612 | 1:1115 - 1:4548 | 1:50 | 1:29400 | |
| | AAV5 | Male | 1:5 | 1:5 - 1:18 | 1:5 | 1:273 | **0.011** |
| | | Female | 1:19 | 1:15 - 1:50 | 1:5 | 1:302 | |
| | AAV6 | Male | 1:37 | 1:15 - 1:70 | 1:5 | 1:1109 | 0.388 |
| | | Female | 1:58 | 1:25 - 1:73 | 1:5 | 1:843 | |
| | AA8 | Male | 1:59 | 1:19 - 1:154 | 1:5 | 1:3724 | 0.356 |
| | | Female | 1:78 | 1:63 - 1:181 | 1:5 | 1:2617 | |
| | AAV9 | Male | 1:144 | 1:50 - 1:242 | 1:50 | 1:7654 | **0.056** |
| | | Female | 1:452 | 1:185 - 1:773 | 1:50 | 1:4686 | |
| | AAV2.7m8 | Male | 1:164 | 1:89 - 1:344 | 1:5 | 1:10240 | 0.231 |
| | | Female | 1:342 | 1:174 - 1:534 | 1:5 | 1:7360 | |
| Healthcare workers | AAV2 | Male | 1:1146 | 1:197 - 1:2914 | 1:50 | 1:28855 | 0.42 |
| | | Female | 1:2402 | 1:915 - 1:3968 | 1:50 | 1:46936 | |
| | AAV5 | Male | 1:26 | 1:5 - 1:50 | 1:5 | 1:232 | 0.255 |
| | | Female | 1:50 | 1:13 - 1:65 | 1:5 | 1:1269 | |
| | AAV6 | Male | 1:33 | 1:5 - 1:94 | 1:5 | 1:319 | 0.21 |
| | | Female | 1:66 | 1:31 - 1:132 | 1:5 | 1:1069 | |
| | AA8 | Male | 1:66 | 1:5 - 1:263 | 1:5 | 1:1383 | 0.57 |
| | | Female | 1:127 | 1:47 - 1:269 | 1:5 | 1:3243 | |

| | | | | | | | |
|---|---|---|---|---|---|---|---|
| | AAV9 | Male | 1:491 | 1:50 - 1:787 | 1:50 | 1:6409 | 0.698 |
| | | Female | 1:298 | 1:123 - 1:792 | 1:50 | 1:4918 | |
| | AAV2.7m8 | Male | 1:364 | 1:16 - 1:532 | 1:5 | 1:4141 | 0.639 |
| | | Female | 1:327 | 1:145 - 1:675 | 1:5 | 1:5704 | |
| RP | AAV2 | Male | 1:1246 | 1:685 - 1:3775 | 1:50 | 1:27487 | 0.756 |
| | | Female | 1:773 | 1:50 - 1:13904 | 1:50 | 1:21360 | |
| | AAV5 | Male | 1:5 | 1:5 - 1:31 | 1:5 | 1:2166 | 0.988 |
| | | Female | 1:5 | 1:5 - 1:70 | 1:5 | 1:256 | |
| | AAV6 | Male | 1:34 | 1:5 - 1:155 | 1:5 | 1:1150 | 0.679 |
| | | Female | 1:17 | 1:5 - 1:152 | 1:5 | 1:593 | |
| | AA8 | Male | 1:57 | 1:17 - 1:197 | 1:5 | 1:2536 | 0.932 |
| | | Female | 1:156 | 1:5 - 1:331 | 1:5 | 1:2774 | |
| | AAV9 | Male | 1:176 | 1:50 - 1:576 | 1:50 | 1:23381 | 0.581 |
| | | Female | 1:320 | 1:50 - 1:1080 | 1:50 | 1:4916 | |
| | AAV2.7m8 | Male | 1:156 | 1:5 - 1:472 | 1:5 | 1:9716 | 0.798 |
| | | Female | 1:97 | 1:5 - 1:977 | 1:5 | 1:4219 | |

HAP, healthy adult participants; RP, retinitis pigmentosa patients; 95% CI, 95% confidence interval.

**Table S2. NAbs characteristics by age groups in adult controls, healthcare workers and RP patients**

| Group | Serotype | Age | Median | 95% CI | Minimum | Maximum | p-value |
|---|---|---|---|---|---|---|---|
| HAP | AAV2 | 18 to 40 | 1:813 | 1:221 - 1:2467 | 1:50 | 1:29400 | **0.031** |
| | | 40 to 60 | 1:3001 | 1:1115 - 1:4350 | 1:50 | 1:28095 | |
| | AAV5 | 18 to 40 | 1:5 | 1:5 - 1:18 | 1:5 | 1:284 | **0.022** |
| | | 40 to 60 | 1:19 | 1:14 - 1:49 | 1:5 | 1:302 | |
| | AAV6 | 18 to 40 | 1:23 | 1:5 - 1:60 | 1:5 | 1:874 | **0.053** |
| | | 40 to 60 | 1:61 | 1:43 - 1:83 | 1:5 | 1:1109 | |
| | AA8 | 18 to 40 | 1:51 | 1:15 - 1:136 | 1:5 | 1:2458 | 0.079 |
| | | 40 to 60 | 1:92 | 1:63 - 1:206 | 1:5 | 1:3724 | |
| | AAV9 | 18 to 40 | 1:144 | 1:50 - 1:242 | 1:50 | 1:5313 | **0.062** |
| | | 40 to 60 | 1:308 | 1:175 - 1:694 | 1:50 | 1:7654 | |
| | AAV2.7m8 | 18 to 40 | 1:114 | 1:11 - 1:284 | 1:5 | 1:6597 | **0.011** |
| | | 40 to 60 | 1:376 | 1:207 - 1:534 | 1:5 | 1:10240 | |
| Healthcare workers | AAV2 | 18 to 40 | 1:1275 | 1:956 - 1:3333 | 1:50 | 1:19825 | 0.813 |
| | | 40 to 60 | 1:1547 | 1:797 - 1:4218 | 1:50 | 1:46936 | |
| | AAV5 | 18 to 40 | 1:41 | 1:15 - 1:50 | 1:5 | 1:305 | 0.667 |
| | | 40 to 60 | 1:50 | 1:5 - 1:162 | 1:5 | 1:1269 | |
| | AAV6 | 18 to 40 | 1:63 | 1:31 - 1:82 | 1:5 | 1:1068 | 0.891 |
| | | 40 to 60 | 1:36 | 1:13 - 1:179 | 1:5 | 1:1069 | |
| | AA8 | 18 to 40 | 1:99 | 1:44 - 1:220 | 1:5 | 1:3243 | 0.449 |
| | | 40 to 60 | 1:72 | 1:12 - 1:663 | 1:5 | 1:1383 | |
| | AAV9 | 18 to 40 | 1:235 | 1:126 - 1:694 | 1:50 | 1:4918 | 0.199 |
| | | 40 to 60 | 1:726 | 1:50 - 1:1631 | 1:50 | 1:6409 | |

| | | | | | | | |
|---|---|---|---|---|---|---|---|
| | AAV2.7m8 | 18 to 40 | 1:265 | 1:129 - 1:532 | 1:5 | 1:3782 | 0.41 |
| | | 40 to 60 | 1:391 | 1:59 - 1:1543 | 1:5 | 1:5704 | |
| RP | AAV2 | 18 to 40 | 1:1067 | 1:50 - 1:14938 | 1:50 | 1:20755 | 0.885 |
| | | 40 to 60 | 1:1093 | 1:411 - 1:4011 | 1:50 | 1:27487 | |
| | AAV5 | 18 to 40 | 1:5 | 1:5 - 1:55 | 1:5 | 1:262 | 0.836 |
| | | 40 to 60 | 1:7 | 1:5 - 1:38 | 1:5 | 1:2166 | |
| | AAV6 | 18 to 40 | 1:57 | 1:5 - 1:218 | 1:5 | 1:683 | 0.486 |
| | | 40 to 60 | 1:18 | 1:5 - 1:101 | 1:5 | 1:1150 | |
| | AA8 | 18 to 40 | 1:92 | 1:5 - 1:282 | 1:5 | 1:2536 | 0.572 |
| | | 40 to 60 | 1:54 | 1:5 - 1:197 | 1:5 | 1:2774 | |
| | AAV9 | 18 to 40 | 1:187 | 1:50 - 1:923 | 1:50 | 1:4495 | 0.729 |
| | | 40 to 60 | 1:95 | 1:50 - 1:873 | 1:50 | 1:23381 | |
| | AAV2.7m8 | 18 to 40 | 1:154 | 1:5 - 1:3613 | 1:5 | 1:7759 | 0.809 |
| | | 40 to 60 | 1:123 | 1:5 - 1:436 | 1:5 | 1:9716 | |

HAP, healthy adult participants; RP, retinitis pigmentosa patients; 95% CI, 95% confidence interval.

**Table S3. Validation of Test Method for AAV2 Neutralizing Antibodies**

| **Method parameters** | **Validation results** |
|---|---|
| **Test item** | Anti-adeno-associated virus type 2 (AAV2) neutralizing antibody |
| **Analytical method** | Cell-based assay based on HEK293T |
| **Matrix** | Normal human serum (Chinese origin) |
| **Minimum required dilution** | 1:50 |
| **Applicable sample** | Human serum samples |
| **Detection instrument** | Microplate reader (Spectra Max L) |
| **Data acquisition software** | SoftMax Pro GxP V5.4.1 |
| **Data processing software** | Microsoft Office Excel (Version 2013) |
| **Titer positive control (TPC)** | |
| TPC1 | 1:50 dilution, 2500 ng/mL (98.9% inhibition) |
| TPC2 | 1:250 dilution, 500 ng/mL (75.4% inhibition) |
| TPC3 | 1:1250 dilution, 100 ng/mL (24.2% inhibition) |
| TPC4 | 1:6250 dilution, 20.0 ng/mL (3.6% inhibition) |
| **Titer negative control** | Pooled serum showed baseline luciferase activity when mixed with AAV-containing cell culture media |
| **Intra-assay precision** | |
| TPC signal CV | 2.7%~32.8% |
| NC signal CV | 1.5%~14.9% |
| TPC titer (NT50) CV | 5.1%~14.2% |
| MSR | 1.160~1.535 |
| **Inter-assay precision** | |
| TPC titer (NT50) CV | 15.2% |
| MSR | 1.428 |
| **Sensitivity** | 379 ng/mL |
| **Selectivity in matrix** | |
| Normal human serum sample | No interference detected |
| Lipaemic sample(300 mg/dL of triglycerides) | No interference detected |
| Haemolysed sample ( 2 % human whole blood) | No interference detected |
| **Drug tolerance** | 2000 ng/mL positive control sample tolerated $1.00\times10^{11}$ vg/mL virus |
| | 1000 ng/mL positive control sample tolerated $1.00\times10^{11}$ vg/mL virus |

| | |
|---|---|
| **Robustness meets acceptance criteria** | |
| Sample incubation time | 1 hour ± 5 minutes |
| Cell incubation time | 24 hours ± 2 hours |
| color development time | 20 minutes ± 5 minutes |
| **Short-term stability** | |
| Room temperature | up to 24.5 hours |
| 2-8°C | up to 72 hours |
| Freeze/thaw (-90 ℃~-60 ℃) | 3 or 6 cycles |
| **Long-term stability** | |
| -25℃~-15℃ | 95 days |
| -90℃~-60℃ | 95 days |
| **Note** | Per USP 40 1106, WRIB white paper and European Bioanalysis Forum consensus: ADA considered stable ≥2 years at ≤-20°C in matrix |

TPC, titer positive control; NC, negative control; CV, coeffcient of variation; MSR, minimum significant ratio; NT50, titers neutralizing 50% of transduction; ADA, anti-drug antibody

**Table S4. Validation of Test Method for AAV5 Neutralizing Antibodies**

| Method parameters | Validation results |
|---|---|
| **Test item** | Anti-adeno-associated virus type 5 (AAV5) neutralizing antibody |
| **Analytical method** | Cell-based assay based on HEK293T |
| **Matrix** | Normal human serum (Chinese origin) |
| **Minimum required dilution** | 1:5 |
| **Applicable sample** | Human serum samples |
| **Detection instrument** | Microplate reader (Spectra Max L) |
| **Data acquisition software** | SoftMax Pro GxP V5.4.1 |
| **Data processing software** | Microsoft Office Excel (Version 2013 or higher) |
| **Titer positive control (TPC)** | |
| TPC1 | 1:5 dilution, 800 ng/mL (98.5% inhibition) |
| TPC2 | 1:20 dilution, 200 ng/mL (73.4% inhibition) |
| TPC3 | 1:80 dilution, 50.0 ng/mL (30.4% inhibition) |
| TPC4 | 1:320 dilution, 12.5 ng/mL (7.8% inhibition) |
| **Titer negative control** | Pooled serum showed baseline luciferase activity when mixed with AAV-containing cell culture media |
| **Intra-assay precision** | |
| TPC signal CV | 2.6%~24.4% |
| NC signal CV | 3.1%~8.1% |
| TPC titer (NT50) CV | 3.0%~12.2% |
| MSR | 1.089~1.407 |
| **Inter-assay precision** | |
| TPC titer (NT50) CV | 9.4% |
| MSR | 1.248 |
| **Sensitivity** | 138 ng/mL |
| **Selectivity in matrix** | |
| Normal human serum sample | No interference detected |
| Lipaemic sample(300 mg/dL of triglycerides) | No interference detected |
| Haemolysed sample ( 2 % human whole blood) | No interference detected |
| **Drug tolerance** | 800 ng/mL positive control sample tolerated $1.00\times10^{11}$ vg/mL virus |
| | 200 ng/mL positive control sample tolerated $1.00\times10^{11}$ vg/mL virus |
| **Robustness meets acceptance criteria** | |

| | |
|---|---|
| Sample incubation time | 1 hour ± 5 minutes |
| Cell incubation time | 24 hours ± 2 hours |
| color development time | NA |
| **Short-term stability** | |
| Room temperature | up to 24 hours |
| 2-8°C | up to 73 hours |
| Freeze/thaw (-90 ℃~-60 ℃) | 3 or 6 cycles |
| **Long-term stability** | |
| -25℃~-15℃ | 108 days |
| -90℃~-60℃ | 108 days |
| **Note** | Per USP 40 1106, WRIB white paper and European Bioanalysis Forum consensus: ADA considered stable ≥2 years at ≤-20°C in matrix |

TPC, titer positive control; NC, negative control; CV, coeffcient of variation; MSR, minimum significant ratio; NT50, titers neutralizing 50% of transduction; ADA, anti-drug antibody

**Table S5. Validation of Test Method for AAV6 Neutralizing Antibodies**

| Method parameters | Validation results |
|---|---|
| **Test item** | Anti-adeno-associated virus type 6 (AAV6) neutralizing antibody |
| **Analytical method** | Cell-based assay based on HEK293T |
| **Matrix** | Normal human serum (Chinese origin) |
| **Minimum required dilution** | 1:5 |
| **Applicable sample** | Human serum samples |
| **Detection instrument** | Microplate reader (Spectra Max L) |
| **Data acquisition software** | SoftMax Pro GxP 7.2 |
| **Data processing software** | Microsoft Office Excel (Version 2016) |
| **Titer positive control (TPC)** | |
| TPC1 | 1:5 dilution, 1000 ng/mL (99.1% inhibition) |
| TPC2 | 1:20 dilution, 250 ng/mL (60.7% inhibition) |
| TPC3 | 1:80 dilution, 62.5 ng/mL (21.2% inhibition) |
| TPC4 | 1:320 dilution, 15.6 ng/mL (8.2% inhibition) |
| **Titer negative control** | Pooled serum showed baseline luciferase activity when mixed with AAV-containing cell culture media |
| **Intra-assay precision** | |
| TPC signal CV | 0.7%~20.9% |
| NC signal CV | 2.4%~5.1% |
| TPC titer (NT50) CV | 2.5%~10.7% |
| MSR | 1.073~1.381 |
| **Inter-assay precision** | |
| TPC titer (NT50) CV | 0.127 |
| MSR | 1.358 |
| **Sensitivity** | 225 ng/mL |
| **Selectivity in matrix** | |
| Normal human serum sample | No interference detected |
| Lipaemic sample(300 mg/dL of triglycerides) | No interference detected |
| Haemolysed sample ( 2 % human whole blood) | No interference detected |
| **Drug tolerance** | 1000 ng/mL positive control sample tolerated $1.00\times10^{11}$ vg/mL virus |
| | 500 ng/mL positive control sample tolerated $1.00\times10^{11}$ vg/mL virus |

| | |
|---|---|
| **Robustness meets acceptance criteria** | |
| Sample incubation time | 1 hour ± 5 minutes |
| Cell incubation time | 24 hours ± 2 hours |
| color development time | NA |
| **Short-term stability** | |
| Room temperature | up to 25 hours |
| 2-8°C | up to 72 hours |
| Freeze/thaw (-90 ℃~-60 ℃) | 3 or 6 cycles |
| **Long-term stability** | |
| -25℃~-15℃ | NA |
| -90℃~-60℃ | NA |
| **Note** | Per USP 40 1106, WRIB white paper and European Bioanalysis Forum consensus: ADA considered stable ≥2 years at ≤-20°C in matrix |

TPC, titer positive control; NC, negative control; CV, coeffcient of variation; MSR, minimum significant ratio; NT50, titers neutralizing 50% of transduction; ADA, anti-drug antibody

**Table S6. Validation of Test Method for AAV8 Neutralizing Antibodies**

| Method parameters | Validation results |
|---|---|
| **Test item** | Anti-adeno-associated virus type 8 (AAV8) neutralizing antibody |
| **Analytical method** | Cell-based assay based on HEK293T |
| **Matrix** | Normal human serum (Chinese origin) |
| **Minimum required dilution** | 1:5 |
| **Applicable sample** | Human serum samples |
| **Detection instrument** | Microplate reader (Spectra Max L) |
| **Data acquisition software** | SoftMax Pro GxP V5.4.1 |
| **Data processing software** | Microsoft Office Excel (Version 2016) |
| **Titer positive control (TPC)** | |
| TPC1 | 1:5 dilution, 200 ng/mL (96.1% inhibition) |
| TPC2 | 1:20 dilution, 50.0 ng/mL (60.0% inhibition) |
| TPC3 | 1:80 dilution, 12.5 ng/mL (17.5% inhibition) |
| TPC4 | 1:320 dilution, 3.13 ng/mL (5.6% inhibition) |
| **Titer negative control** | Pooled serum showed baseline luciferase activity when mixed with AAV-containing cell culture media |
| **Intra-assay precision** | |
| TPC signal CV | 2.0%~18.7% |
| NC signal CV | 1.8%~6.7% |
| TPC titer (NT50) CV | 4.2%~20.2% |
| MSR | 1.127~1.937 |
| **Inter-assay precision** | |
| TPC titer (NT50) CV | 16.5% |
| MSR | 1.525 |
| **Sensitivity** | 48.2 ng/mL |
| **Selectivity in matrix** | |
| Normal human serum sample | No interference detected |
| Lipaemic sample(300 mg/dL of triglycerides) | No interference detected |
| Haemolysed sample ( 2 % human whole blood) | No interference detected |
| **Drug tolerance** | 200 ng/mL positive sample tolerated up to $1.00 \times 10^{11}$ vg/mL drug |
| | 100 ng/mL positive sample tolerated up to $1.00 \times 10^{10}$ vg/mL drug |

| | |
|---|---|
| **Robustness meets acceptance criteria** | |
| Sample incubation time | 1 hour ± 5 minutes |
| Cell incubation time | 24 hours ± 2 hours |
| color development time | NA |
| **Short-term stability** | |
| Room temperature | up to 28 hours 7 minutes |
| 2-8°C | up to 76 hours 8 minutes |
| Freeze/thaw (-90 ℃~-60 ℃) | 3 or 6 cycles |
| **Long-term stability** | |
| -25℃~-15℃ | 112 days |
| -90℃~-60℃ | 112 days |
| **Note** | Per USP 40 1106, WRIB white paper and European Bioanalysis Forum consensus: ADA considered stable ≥2 years at ≤-20°C in matrix |

TPC, titer positive control; NC, negative control; CV, coeffcient of variation; MSR, minimum significant ratio; NT50, titers neutralizing 50% of transduction; ADA, anti-drug antibody

**Table S7. Validation of Test Method for AAV9 Neutralizing Antibodies**

| Method parameters | Validation results |
|---|---|
| **Test item** | Anti-adeno-associated virus type 9 (AAV9) neutralizing antibody |
| **Analytical method** | Cell-based assay based on HEK293T |
| **Matrix** | Normal human serum (Chinese origin) |
| **Minimum required dilution** | 1:50 |
| **Applicable sample** | Human serum samples |
| **Detection instrument** | Microplate reader (Spectra Max L) |
| **Data acquisition software** | SoftMax Pro GxP V5.4.1 |
| **Data processing software** | Microsoft Office Excel (Version 2013 or higher) |
| **Titer positive control (TPC)** | |
| TPC1 | 1:50 dilution, 2000 ng/mL (98.5% inhibition) |
| TPC2 | 1:250 dilution, 400 ng/mL (76.1% inhibition) |
| TPC3 | 1:1250 dilution, 80.0 ng/mL (30.6% inhibition) |
| TPC4 | 1:6250 dilution, 16.0 ng/mL (4.6% inhibition) |
| **Titer negative control** | Pooled serum showed baseline luciferase activity when mixed with AAV-containing cell culture media |
| **Intra-assay precision** | |
| TPC signal CV | 3.3%~21.6% |
| NC signal CV | 4.0%~9.4% |
| TPC titer (NT50) CV | 4.1%~17.0% |
| MSR | 1.124~1.601 |
| **Inter-assay precision** | |
| TPC titer (NT50) CV | 12.9% |
| MSR | 1.346 |
| **Sensitivity** | 279 ng/mL |
| **Selectivity in matrix** | |
| Normal human serum sample | No interference detected |
| Lipaemic sample(300 mg/dL of triglycerides) | No interference detected |
| Haemolysed sample ( 2 % human whole blood) | No interference detected |
| **Drug tolerance** | 2000 ng/mL positive control sample tolerated $1.00\times10^{11}$ vg/mL virus |
| | 1000 ng/mL positive control sample tolerated $1.00\times10^{11}$ vg/mL virus |

| | |
|---|---|
| **Robustness meets acceptance criteria** | |
| Sample incubation time | 1 hour ± 5 minutes |
| Cell incubation time | 22 hours ± 2 hours |
| color development time | NA |
| **Short-term stability** | |
| Room temperature | up to 26 hours |
| 2-8°C | up to 74 hours |
| Freeze/thaw (-90 ℃~-60 ℃) | 3 or 6 cycles |
| **Long-term stability** | |
| -25℃~-15℃ | NA |
| -90℃~-60℃ | NA |
| **Note** | Per USP 40 1106, WRIB white paper and European Bioanalysis Forum consensus: ADA considered stable ≥2 years at ≤-20°C in matrix |

TPC, titer positive control; NC, negative control; CV, coeffcient of variation; MSR, minimum significant ratio; NT50, titers neutralizing 50% of transduction; ADA, anti-drug antibody

**Table S8. Validation of Test Method for AAV2.7m8 Neutralizing Antibodies**

| **Method parameters** | **Validation results** |
|---|---|
| **Test item** | Anti-adeno-associated virus type 2 mutation (AAV2.7m8) neutralizing antibody |
| **Analytical method** | Cell-based assay based on HEK293T |
| **Matrix** | Normal human serum (Chinese origin) |
| **Minimum required dilution** | 1:5 |
| **Applicable sample** | Human serum samples |
| **Detection instrument** | Microplate reader (Spectra Max L) |
| **Data acquisition software** | SoftMax Pro GxP V5.4.1 |
| **Data processing software** | Microsoft Office Excel (Version 2016) |
| **Titer positive control (TPC)** | |
| TPC1 | 1:5 dilution, 800 ng/mL (97.9% inhibition) |
| TPC2 | 1:10 dilution, 400 ng/mL (85.7% inhibition) |
| TPC3 | 1:20 dilution, 200 ng/mL (53.6% inhibition) |
| TPC4 | 1:40 dilution, 100 ng/mL (26.1% inhibition) |
| **Titer negative control** | Pooled serum showed baseline luciferase activity when mixed with AAV-containing cell culture media |
| **Intra-assay precision** | |
| TPC signal CV | 3.5%~22.9% |
| NC signal CV | 2.8%~7.5% |
| TPC titer (NT50) CV | 4.8%~13.5% |
| MSR | 1.148~1.481 |
| **Inter-assay precision** | |
| TPC titer (NT50) CV | 15.7% |
| MSR | 1.451 |
| **Sensitivity** | 224 ng/mL |
| **Selectivity in matrix** | |
| Normal human serum sample | No interference detected |
| Lipaemic sample (300 mg/dL of triglycerides) | No interference detected |
| Haemolysed sample ( 2 % human whole blood) | No interference detected |
| **Drug tolerance** | 800 ng/mL positive samples can tolerate up to $1.00\times10^{10}$ gc/mL drug |
| | 400 ng/mL positive samples can tolerate up to $1.00\times10^{9}$ gc/mL drug |

| | |
|---|---|
| **Robustness meets acceptance criteria** | |
| Sample incubation time | 1 hour ± 5 minutes |
| Cell incubation time | 24 hours ± 2 hours |
| color development time | NA |
| **Short-term stability** | |
| Room temperature | up to 25.5 hours |
| 2-8°C | up to 72.5 hours |
| Freeze/thaw (-90 ℃~-60 ℃) | 3 or 6 cycles |
| **Long-term stability** | |
| -25℃~-15℃ | NA |
| -90℃~-60℃ | NA |
| **Note** | Per USP 40 1106, WRIB white paper and European Bioanalysis Forum consensus: ADA considered stable ≥2 years at ≤-20°C in matrix |

TPC, titer positive control; NC, negative control; CV, coeffcient of variation; MSR, minimum significant ratio; NT50, titers neutralizing 50% of transduction; ADA, anti-drug antibody

**Table S9. MOI and baseline luciferase activity in transduction inhibition-based NAb assays for each NAb serotype.**

| NAb serotype | AAV2 | AAV5 | AAV6 | AAV8 | AAV9 | AAV2.7m8 |
|---|---|---|---|---|---|---|
| AAV infection MOI (vg/cell) | 1500 | 16000 | 16000 | 10000 | 12400 | 1000 |
| Baseline signal (RLU, NC from method validation) | 98699~ 234346 | 1126342~1704036 | 3508517~ 10143014 | 2588869~ 5055186 | 111575~ 265109 | 2392807~3824444 |

MOI, multiplicity of infection; vg, viral genomes; RLU, relative light units; NC, negative control; AAV, adeno-associated virus; NAbs, neutralizing antibodies.

**Supplemental Methods**

**Pseudo-Code for the Entire Feature Selection and Model Training**

Algorithm: Recursive Feature Elimination for LightGBM Regression

Input:

- X_train, y_train: Training dataset features and target
- X_test, y_test: Test dataset features and target
- n_features_to_select: Minimum number of features (default: 1)
- step: Number of features to remove per iteration (default: 1)
- cv: Number of cross-validation folds (default: 5)
- lgbm_params: LightGBM hyperparameters

Output:

- best_features: List of selected feature names
- results_df: Performance metrics for each iteration
- cv_tables: Cross-validation RMSE and $R^2$ tables
- shap_values: SHAP values for feature interpretation

Procedure:

1. Initialize:
    - num_features ← total number of features
    - selected_features ← boolean array (all True)
    - results ← empty list

2. Set LightGBM parameters:
    - objective: 'regression'
    - num_leaves: 31
    - learning_rate: 0.05
    - n_estimators: 100

- random_state: 42

3. For each iteration (from num_features down to 1, step by step):

   a. Select current features:

      X_train_selected ← X_train[:, selected_features]

      X_test_selected ← X_test[:, selected_features]

   b. Perform K-Fold Cross-Validation:

      For each fold in cv:

         - Split training data into train_fold and validation_fold
         - Train LightGBM model on train_fold
         - Predict on validation_fold
         - Calculate RMSE and $R^2$ for validation_fold
         - Store fold results

      - Calculate mean_RMSE, std_RMSE, SE_RMSE across folds
      - Calculate mean_$R^2$, std_$R^2$, SE_$R^2$ across folds

   c. Train model on full training set:

      - Fit LightGBM on X_train_selected
      - Predict on X_test_selected
      - Calculate test_RMSE and test_$R^2$

   d. Store iteration results:

      - n_features, CV metrics, test metrics, selected_features

   e. Feature elimination:

- Extract feature_importances from trained model
    - Identify least important feature
    - Set selected_features[least_important_idx] ← False
    - Remove least important feature

4. Identify optimal feature subset:
   - best_idx ← argmin(CV_RMSE_mean) across all iterations
   - best_features ← features corresponding to best_idx
   - Note: All features evaluated down to n=1 to capture complete
     performance curve and identify global minimum

5. Train final model with optimal features:
   a. Model training:
      - Fit LightGBM on X_train[best_features]
      - Evaluate on X_test[best_features]
      - Calculate final RMSE, MAE, $R^2$

   b. Feature interpretation using SHAP:
      - Initialize TreeExplainer with trained model
      - Calculate SHAP values for X_train[best_features]
      - Generate SHAP summary plot to visualize:
        * Feature contribution magnitude
        * Feature value impact on predictions
        * Relative importance of selected features

6. Return:
   - best_features (optimal feature subset)
   - results_df (performance metrics across all iterations)

- cv_rmse_table, cv_r2_table (detailed cross-validation results)
- shap_values (feature contribution analysis)
