# Supplementary material for: Seroprevalence of anti-AAV neutralizing antibodiesin healthy and retinitis pigmentosa cohorts: A multi-province study in China
Source: Mol Ther Adv. 2026 Apr 28;34(2):201743. doi: 10.1016/j.omta.2026.201743 (PMC13254674; doi:10.1016/j.omta.2026.201743)
Supplement: Document S2. Article plus supplemental information [file mmc2.pdf]

Molecular Therapy
Advances
Original Article

Seroprevalence of anti-AAV neutralizing antibodies in healthy and retinitis pigmentosa cohorts: A multi-province study in China

Jingxiao Du,[1,2,3,4,5,9] Huixun Jia,[1,2,3,4,5,9] Xuewei Xie,[6,7,9] Xiaosa Li,[1,2,3,4,5] Ting Zhang,[1,2,3,4,5] Yidong Wu,[1,2,3,4,5] Zishi Liu,[1,2,3,4,5] Haoliang Zhang,[1,2,3,4,5] Zhiyan Li,[8] Weihua Gu,[8] Xinkun Tao,[8] Jieqiong Chen,[1,2,3,4,5] Junran Sun,[1,2,3,4,5] Tong Li,[1,2,3,4,5] and Xiaodong Sun[1,2,3,4,5]

[1]Department of Ophthalmology, Shanghai General Hospital, Shanghai Jiao Tong University School of Medicine, Shanghai 200080, China; [2]National Clinical Research Center for Ophthalmic Diseases, Shanghai 200080, China; [3]Shanghai Engineering Center for Visual Science and Photomedicine, Shanghai 200080, China; [4]Shanghai Gene Therapy Center, Shanghai 200080, China; [5]Shanghai Key Laboratory of Fundus Diseases, Shanghai 200080, China; [6]China National Clinical Research Center for Neurological Diseases, Beijing 100070, China; [7]Department of Neurology, Beijing Tiantan Hospital, Capital Medical University, Beijing 100070, China; [8]Shanghai Xihua Scientific Co.Ltd, Shanghai 200000, China

Anti-AAV neutralizing antibody (NAb) level s vary widely across regions and serotypes, yet current data on their seroprevalence in the Chinese population remain limited. This multi-provincial observational study recruited 286 adult participants across six Chinese provinces to assess NAbs against six clinically relevant serotypes using transduction inhibition assays. Results showed widespread NAb prevalence in the Chinese population, with anti-AAV2 having the highest median titer (1:1,675) and anti-AAV5 the lowest (1:15). Prevalence patterns aligned with capsid sequence conservation, with higher similarity serotypes showing comparable prevalence. Geographically, southern provinces showed significantly greater seroprevalence of anti-AAV6 and anti-AAV8 NAbs than northern provinces. Healthcare workers performing invasive procedures had higher titers for anti-AAV5 NAb than staff performing non-invasive procedures. Tree-based regression revealed serotype-specific correlates: total cholesterol and HBsAb showed broad statistically positive associations with NAb titers, while ApoA1 was selectively positive and ApoB negative. This work provides real-world reference data for NAb prevalence in China, demonstrating widespread prevalence with particularly high AAV2 titers influenced by geographical and demographic factors. Considering the hepatotropic nature of AAV and the importance of hepatic lipid metabolism, the correlation between NAb titers and lipid profiles (cholesterol and apolipoproteins) points to potential biological mechanisms that merit further study.

INTRODUCTION

Adeno-associated virus (AAV) vectors are widely used as gene delivery tools for the treatment of a wide range of genetic and acquired diseases. At least six AAV-based gene therapy products have received regulatory approval by the US Food and Drug Administration (FDA), pioneered by the ocular gene therapy-Luxturna in 2017.[1] Nevertheless, viral gene transfer efficiency and the durability of transgene expression of AAV-based agents may be compromised by pre-existing neutralizing antibodies (NAbs), which are generated as part of the adaptive immune response following exposure to wild-type viral capsids or cross-reactive AAV serotypes.[2] Even relatively low titers of NAbs can completely neutralize high doses of therapeutic vectors and significantly impair transgene expression following systemic vector delivery, making the immunological barrier one of the most pressing limitations in AAV-based therapies.[3] Preexisting anti-AAV8 NAbs at titers exceeding 1:10 have been shown to significantly reduce hepatocyte transduction efficiency in macaques, whereas in clinical studies, NAbs at a pretreatment titer of 1:17 severely impaired Factor IX expression in the patient receiving AAV-Factor IX treatment.[4,5] This humoral immunity characterized by NAbs against wild-type AAVs poses an obstacle to effective systemic gene delivery using AAV vectors.

As of this writing, over 300 AAV-based gene therapy trials are registered on clinicaltrials.gov but the usage of NAb screening varies greatly by disease indications and route of drug administration.[6,7] Although over half of the registered trials introduce NAb screening for patient enrollment, fewer than one-fourth of the actively

Received 31 October 2025; accepted 24 April 2026;
https://doi.org/10.1016/j.omta.2026.201743.

[9]These authors contributed equally

Correspondence: Tong Li, National Clinical Research Center for Ophthalmic Diseases, Department of Ophthalmology, Shanghai General Hospital, Shanghai Jiao Tong University, School of Medicine, 100 Haining Road, Shanghai 200080, China.
E-mail: litong_linda@outlook.com

Correspondence: Xiaodong Sun, National Clinical Research Center for Ophthalmic Diseases, Department of Ophthalmology, Shanghai General Hospital, Shanghai Jiao Tong University, School of Medicine, Shanghai Key Laboratory of Fundus Diseases, 100 Haining Road, Shanghai 200080, China.
E-mail: xdsun@sjtu.edu.cn

Published by Elsevier Inc. on behalf of The American Society of Gene and Cell Therapy.
This is an open access article under the CC BY license (http://creativecommons.org/licenses/by/4.0/).

Table 1. Demographic characteristics of participants

		Healthy adult participants	Healthcare workers	Retinitis pigmentosa	Total
Number of participants		185	61	40	286
Sex (male:female)		1.72	0.56	1.67	1.34
Age (mean ± SD, years)		39.9 ± 11.9	37.3 ± 7.4	37.3 ± 14.2	39.7 ± 14.9
Age distribution (%)−−	6–18	0	0	10	1.4
	18–40	48.11	65.57	40.00	50.7
	40–60	51.89	34.43	50.00	47.9

recruiting studies (e.g., NCT05152732 and NCT05302271) specify NAb thresholds (cutoffs varied from 1:1 to 1:1,200) to determine patient eligibility.[6] Poorly standardized serological assays for determining NAb levels, along with variations in methodology, make NAb titers across different studies incomparable and may contribute to heterogeneity in therapeutic outcomes following AAV administration in the presence of pre-existing NAbs.[8–10]

Reported NAb prevalence varies widely—from fewer than 10% to over 90%—depending on the AAV serotype and geographic region.[11] These differences are influenced by factors such as prior exposure to wild-type AAV, regional epidemiology, and serotype-specific capsid characteristics.[8] A clear understanding of anti-AAV NAb prevalence across different populations is essential for the rational design of clinical trials, the development of optimized AAV vectors, and the determination of delivery strategies. To assess the seroprevalence of anti-AAV NAbs, a highly sensitive, cell-based transduction inhibition (TI) assay has been developed.[12,13] This assay quantifies NAbs by pre-incubating serially diluted patient serum with rAAV vectors carrying reporter genes, followed by measurement of reductions in reporter gene expression. This approach allows the direct detection of NAbs and non-antibody neutralizing factors that impact transduction, providing an accurate assessment of resistance to AAV capsids in the plasma, typically expressed as the ID50 (the serum dilution at which 50% of transduction is inhibited).[14] However, the stringent experimental procedures, the high costs, and the need for specialized laboratories make large-scale testing and commercial assay kit development challenging.[2]

To date, only a few studies have screened the seroprevalence of specific AAV serotypes in certain regions of China using validated TI assays.[15,16] This observational study systematically presents the seroprevalence and ID50-basedNAb titers against AAV2, AAV5, AAV6, AAV8, AAV9, and AAV2.7m8 (an AAV2-based capsid variant generated through directed evolution) in representative healthy individuals, healthcare workers, and patients with retinitis pigmentosa (RP), who are at the forefront of ocular rAAV gene therapy, across six provinces in China. Furthermore, we performed an exploratory tree-based machine learning analysis to identify routine physiological parameters associated with anti-AAV NAb titers across multiple serotypes. The results will facilitate vector optimization and the design of future gene therapy trials and refine the identification of eligible populations for broader implementation of AAV-based therapies.

RESULTS

Demographic characteristics of the participants

A total of 286 participants were enrolled in this study, including 185 healthy adult participants (HAPs), 40 patients with RP, and 61 healthcare workers from Shanghai General Hospital (Table 1). Adult participants were recruited from Shanghai (30), Beijing (30), Hunan (30), Guangdong (30), Shandong (30), and Harbin (35), with a male-to-female ratio of 1.72 and a mean age of 39.9 ± 11.9 years. The northern provinces include Beijing, Shandong, and Harbin, while the southern provinces include Shanghai, Hunan, and Guangdong. The gender distribution differed significantly between the northern and southern regions, with a higher proportion of males in the southern group compared to the northern group (74.4% vs. 52.6%, p = 0.003). The age distribution did not differ significantly between the two regions, with participants aged 40–60 years accounting for 54.7% of the northern group and 48.9% of the southern group (p = 0.517). At the province level, the distributions of age and gender varied significantly across provinces (chi-square test: gender, $p < 0.001$; age, $p = 0.012$).

Among the 61 healthcare workers, 23 were identified with a high frequency of invasive procedures, and 38 were considered to work under non-invasive scenarios, with male-to-female ratios of 0.77 and 0.46, and mean ages of 39.2 ± 7.7 and 36.1 ± 7.1 years, respectively. The gender distribution did not differ significantly between the invasive and non-invasive groups (43.5% male vs. 31.6% male, $p = 0.507$), nor did the age distribution (39.1% aged 40–60 in the invasive vs. 31.6% in the non-invasive group, $p = 0.746$). The RP group presented with a male-to-female ratio of 1.67 and mean age of 37.3 ± 14.2 years (Table 1).

Prevalence of anti-AAV NAb seroprevalence in healthy adults: Serotype differences and age- and gender-associated trends

This study assessed NAb seroprevalence across serial serum dilutions for six AAV serotypes (AAV2, AAV5, AAV6, AAV8, AAV9, and AAV2.7m8) in healthy participants and designated subgroups, stratified by demographic characteristics. In healthy participants, AAV2 showed the highest NAb prevalence, followed by AAV9 and AAV2.7m8, while AAV5 exhibited the lowest prevalence.

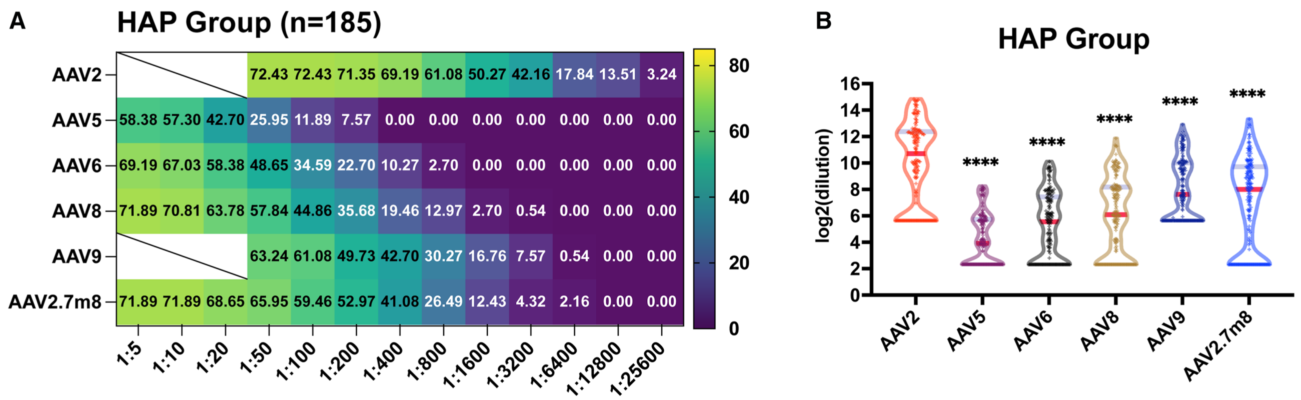

Figure 1. Prevalence and titers of anti-AAV NAbs in HAP group

(A) Heatmap of anti-AAV NAbs seroprevalence across different dilution levels in HAP group for AAV2, AAV5, AAV6, AAV8, AAV9, and AAV2.7m8. The percentage of seropositive individuals is indicated by color intensity according to the scale, with numbers inside squares representing the exact proportion at each serotype and dilution level. (B) Truncated violin plots showing the relative levels of anti-AAV NAb titers across different serotypes in the HAP group. NAb titers (ID50) are presented on a log2 scale, with transformed values for each individual displayed as scatter points overlaid on violin plots. Blue lines: quartile values; red lines: median values. Statistical comparisons between groups were performed using the Kruskal-Wallis test followed by Dunn's multiple comparisons test. ****, $p < 0.0001$ compared to AAV2.

Specifically, at a 1:50 serum dilution, the NAb prevalence was highest for AAV2 (72.43%), followed by AAV2.7m8 (65.95%), AAV9 (63.24%), AAV8 (57.84%), AAV6 (48.65%), and AAV5 (25.95%) (Figure 1A). Notably, AAV2 demonstrated substantially higher NAb titers, with 42.16% of participants still showing detectable NAbs at a 1:3,200 dilution, and 3.24% remaining positive even at a 1:25,600 dilution, whereas NAb activity against AAV5 was undetectable in all participants beyond a 1:400 dilution (Figure 1A). These titer levels accounted for the observed prevalence patterns. Among all serotypes, AAV2 exhibited the highest median NAb titers. The median NAb titer for AAV2 was 1:1,675 (1:979 to 1:3061), compared to 1:15 (1:10 to 1:23) for AAV5 in the HAP group (Figure 1B).

In the HAP group, females exhibited higher NAb prevalence than males across AAV serotypes and serum dilutions. For instance, anti-AAV5 NAbs were detected as positive in 75% of females compared with 48.72% in males at a 1:5 dilution, and anti-AAV9 NAbs were found in 73.13% compared with 58.12% at a 1:50 dilution (Figure S1A). Median titers were higher in females for NAbs against AAV5 (1:19 vs. 1:5, $p = 0.011$) and AAV9 (1:452 vs. 1:144, $p = 0.056$). Similar trends with gender were observed in other serotypes, though not statistically significant (Table S1).

Age-related analyses in the HAP group revealed significant or borderline-significant positive correlations between anti-AAV NAb titers and participant age across all serotypes (Figure S2), suggesting an age-dependent increase. Age-stratified descriptive statistics of NAb titers were concordant with the regression results, as heatmaps demonstrated higher NAb prevalence across all dilutions in healthy participants aged 40–60 years compared to those aged 18–40 years (Figure S3A). Median titers were significantly higher in the 40–60 years age group for anti-AAV2 (1:3,001 vs. 1:813, $p = 0.031$), anti-AAV5 (1:19 vs. 1:5, $p = 0.022$), and anti-AAV2.7m8 (1:376 vs. 1:114, $p = 0.011$). Other serotypes showed similar trends toward higher titers in elderly participants, although the differences did not reach statistical significance (Table S2).

Geographical distribution of anti-AAV NAb titers reveals regional differences

To further characterize NAb titers, we calculated descriptive statistics for six AAV serotypes in six provinces across China and performed Ward.D2 unsupervised clustering. Clustering revealed that Guangdong and Hunan, both southern provinces at lower latitudes, exhibited similar titer characteristics; while Beijing, Shandong, and Harbin, northern provinces at higher altitudes, formed another cluster (Figure 2A). Comparisons between southern and northern provinces showed significantly higher titers in the south for AAV6 (1:203 [1:111–1:243] vs. 1:70 [1:55–1:93], $p = 0.0039$), AAV8 (1:264 [1:154–1:322] vs. 1:165 [1:79–1:231], $p = 0.0{,}325$), and AAV2.7m8 (1:686 [1:456–1:1,160] vs. 1:406 [1:284–1:597], $p = 0.0411$). AAV2 (1:5,008 [1:3,397–1:5,634] vs. 1:3,327 [1:2,150–1:4,545], $p = 0.2{,}392$) and AAV5 (1:56 [1:44–1:71] vs. 1:39 [1:18–1:62], $p = 0.1{,}055$) titers were also higher in the south, though the differences did not reach statistical significance (Figure 2B).

To account for potential confounding by age and gender, we constructed multivariable logistic regression models to assess the contributions of age, gender, and geographic region to NAb titers in the HAP population (Table 2). Across all serotype-specific models, the VIF (variance inflation factors) ranged from 1.05 to 1.10 for region, 1.01–1.03 for age, and 1.05–1.10 for gender, indicating no significant multicollinearity among the variables. After adjusting for age and gender, participants from southern regions showed significantly higher NAb titers for AAV2 ($p = 0.049$), AAV6 ($p = 0.001$), and AAV8 ($p = 0.033$) compared with participants from northern regions. In contrast, neither age nor gender was significantly associated

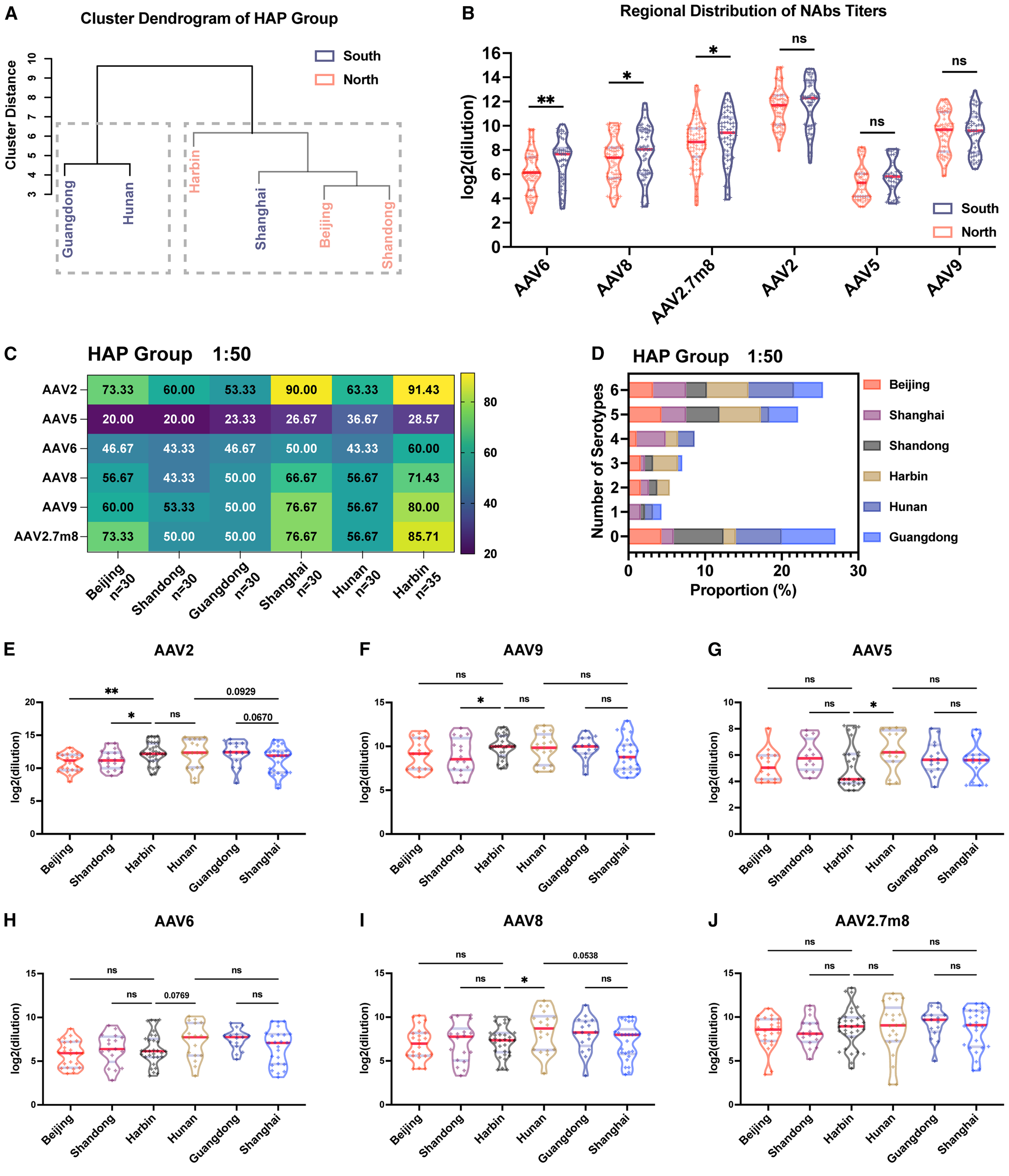

Figure 2. Geographic distribution characteristics of anti-AAV NAbs in HAP group

(A) Hierarchical clustering dendrogram showing NAb characteristics across provinces in the HAP group. The dendrogram integrates NAb titer profiles of all AAV serotypes, clustering provinces with similar NAb titer distribution patterns. Two distinct clusters are identified and marked with different colored dashed boxes. North and South regions are marked with different colors. (B) Truncated violin plots showing the relative levels of NAbs titers between North and South regions in HAP group. (C) Heatmap of AAV NAbs

(legend continued on next page)

with NAb titers for any serotype (all $p > 0.05$). These findings suggest that geographic region (south vs. north) was the predominant factor associated with NAb titer differences in the HAP group.

We further evaluated seroprevalence at the 1:50 dilution across provinces in the HAP group. Harbin and Shanghai exhibited the highest prevalence, with AAV2 NAb prevalence reaching 91.43% and 90.00%, respectively, whereas Guangdong showed the lowest prevalence at 53.33%. Similarly, for AAV2.7m8, AAV9, and AAV8, prevalence was highest in Harbin (85.71%, 80%, and 71.43%, respectively) and lowest in Guangdong (50% for each serotype) (Figure 2C). Further analysis revealed a polarized distribution, with 27.03% of the HAP group positive for all six NAbs and 25.40% completely negative, far exceeding the proportion with partial positivity (1–4 serotypes) (Figure 2D). In the fully negative group, participants were least represented in Shanghai and Harbin (1.62%) and most represented in Hunan, Guangdong, and Shandong (5.95%, 7.03%, 6.49%). In the fully positive group, Shandong and Beijing had the lowest proportions (2.7%, 3.24%), while Shanghai, Hunan, and Guangdong had the highest proportions (4.32%, 5.95%, 3.78%).

Province-level comparisons of NAb titers also revealed distinct deviations from regional patterns. In Harbin, the highest-latitude location of the six provinces, median titers of AAV2 were significantly higher than in Beijing (p = 0.0039) and Shandong (p = 0.0463), and AAV9 titers were also elevated relative to these two lower-latitude northern cities (p = 0.1101 vs. Beijing, p = 0.0468 vs. Shandong) (Figures 2E and 2F). In contrast, NAb titers against AAV5, AAV6, and AAV8 in Harbin were lower than in Hunan (p = 0.0205, 0.0769, 0.0430, respectively) and aligned more closely with those in Beijing and Shandong (Figures 2G–2I). In Shanghai, some serotypes exhibited lower titers compared to other southern cities. The median AAV2 NAb titer in Shanghai (1:3,795 [1:665–1:5,595]) was lower than in Hunan (1:5,185 [1:1,097–1:21,469], p = 0.0929) and Guangdong (1:5,480 [1:2,288–1:13,970], p = 0.0670) (Figure 2E). Similarly, the median AAV8 NAb titer in Shanghai (1:253 [1:63–1:297]) was lower than in Hunan (1:417 [1:79–1:970], p = 0.0538) (Figure 2I). These findings indicate that the seroprevalence of anti-AAV NAbs varies by geographic origin.

Elevated AAV NAb titers among healthcare workers performing invasive procedures

At a 1:50 dilution, AAV2 exhibited the highest prevalence (81.97%), while AAV5 showed the lowest (34.43%) (Figure 3A). Across all dilution levels, the NAb prevalence for the six AAV serotypes was higher in healthcare workers than in the HAP group (Figures 1A and 3A). Specifically, NAb prevalence at a 1:50 dilution for AAV2, AAV9, and AAV2.7m8 was 81.97%, 70.49%, and 73.77% in healthcare workers, respectively, compared with 72.43%, 63.24%, and 65.95% in HAP. For AAV5, AAV6, and AAV8, the prevalence was 34.43% (25.95% in HAP), 54.10% (48.65% in HAP), and 62.30% (57.8% in HAP), respectively (Figures 1A and 3A). Notably, NAbs against AAV5 remained detectable up to a 1:800 dilution in healthcare workers compared with 1:200 in HAP (Figure 3A). Among healthcare workers, anti-AAV2 NAb titers remained high, paralleling the high prevalence. The median titer against AAV2 was 1:1,418 (1:956–1:3259), whereas that against AAV5 was 1:49 (1:17–1:50) (Figure 3B) Similar to the HAP group, the prevalence heatmap in healthcare workers showed an increase across serotypes in females and older age groups (Figures S1B and S3B), but median titers did not differ significantly (Table S1 and S2).

Health workers were subsequently stratified by exposure to invasive procedures, with 37.7% reporting a high frequency of invasive procedures (Figure 3C). At a 1:50 dilution, healthcare workers exposed to invasive procedures displayed higher NAb levels across all six serotypes compared with staff performing non-invasive procedures. For instance, the AAV2 positivity rate was 95.65% in the invasive group, compared to 73.68% in the non-invasive group (Figure 3D). Co-prevalence analysis showed significantly more individuals positive for all six serotypes (39.13% vs. 28.95%) and significantly fewer individuals negative for all six (4.35% vs. 26.32%) in the invasive group (Figure 3E).

In unadjusted analysis, the invasive group exhibited higher median NAb titers across all serotypes compared to the non-invasive group. Specifically, the invasive group demonstrated elevated median NAb titers for AAV5 (1:50 [1:17–1:53], $p < 0.05$), AAV8 (1:169 [1:22–1:259]), and AAV9 (1:438 [1:123–1:740]) compared to the non-invasive group (AAV5: 1:15 [1:5–1:49], AAV8: 1:61 [1:5–1:182], and AAV9: 1:172 [1:50–1:563]) (Figure 3F).

To distinguish the independent contributions of procedure type, age, and gender to NAb titer variations in the healthcare worker cohort, we assessed potential confounding among these variables and constructed multivariable logistic regression models with NAb titers dichotomized at the median, as performed for the HAP group (Table 2). Variance inflation factor (VIF) analysis showed no multicollinearity among predictors, with VIF values ranging from 1.01 to

seroprevalence at 1:50 dilution across different provinces in HAP group. The percentage of seropositive individuals is indicated by color intensity according to the scale, with numbers inside squares representing the exact proportion at each serotype and dilution level. (D) Co-prevalence distribution of NAbs at 1:50 dilution across different provinces in the HAP group. The y axis represents the degree of co-prevalence, ranging from negative NAb activity for all serotypes (0), partial positive for some serotypes (1–5), to positive for all serotypes (6). The x axis shows the proportion (%) of individuals at each co-prevalence level within each province. (E–J) Truncated violin plots showing the relative levels of anti-AAV NAbs titers across different regions (Beijing, Shandong, Guangdong, Shanghai, Hunan, and Harbin). For truncated violin plots, NAb titers (ID50) are presented on a log2 scale, with transformed values for each individual displayed as scatter points overlaid on violin plots. Blue lines: quartile values; red lines: median values. Statistical comparisons between groups were performed using the Kruskal-Wallis test followed by Dunn's multiple comparisons test. *, $p < 0.05$. **, $p < 0.01$. ***, $p < 0.001$. ns, no significance.

Table 2. Associations of geographic region and demographic characteristics with NAb titers against six AAV serotypes based on multivariable logistic regression

–		Comparison–	AAV2		AAV5		AAV6		AAV8		AAV9		AAV2.7m8	
			Coef.	p value	Coef.	p value	Coef.	p value	Coef.	p value	Coef.	p value	Coef.	p value
Healthy adult participants––	region	south: north	0.72	0.049[b]	0.72	0.088[c]	1.23	0.001[a]	0.78	0.033[b]	−0.07	0.853	0.64	0.084[c]
	age distribution	(40 to 60):(18 to 40)	0.08	0.821	0.16	0.691	−0.06	0.882	0.10	0.773	0.17	0.644	0.08	0.826
	gender	male:female	−0.44	0.245	0.51	0.222	0.02	0.955	0.26	0.489	−0.12	0.751	−0.20	0.597
Healthcare workers––	procedure	non-invasive:invasive	−0.12	0.82	−1.07	0.06[c]	−0.18	0.744	−0.14	0.793	−0.61	0.267	−0.42	0.432
	age distribution	(40 to 60):(18 to 40)	0.12	0.82	0.1	0.858	−0.15	0.789	−0.17	0.751	0.64	0.25	0.12	0.821
	gender	male:female	−0.36	0.502	−0.82	0.157	−0.63	0.248	−0.34	0.534	0.11	0.848	−0.06	0.913

Variables within each group were used for logistic regression. Comparisons (A:B) reflect levels within each variable. Positive/negative coefficients indicate higher/lower anti-AAV NAb titers in A relative to B at the corresponding serotype, adjusting for other variables. *p* values indicate the significance of each comparison. RP, retinitis pigmentosa; Coef., coefficient; NAb, neutralizing antibody; AAV, adeno-associated virus.

[a] $p < 0.01$.
[b] $p < 0.05$.
[c] $p < 0.1$.

1.05 for procedure type, 1.01–1.02 for age, and 1.02–1.06 for gender across all serotype models (all VIF <2). After adjustment for age and gender, the association between procedure type and NAb titers did not reach statistical significance. Nevertheless, AAV5 showed a near-significant difference ($p = 0.06$) (Table 2), with the model indicating higher median anti-AAV5 NAb titers in the invasive group compared to the non-invasive group. This trend followed a similar pattern to the unadjusted comparisons (Figure 3F). Although procedure type did not reach statistical significance, regression coefficients were consistently negative, suggesting a trend toward higher anti-AAV NAb titers in departments performing more invasive procedures. Neither age nor gender showed a significant association with NAb titers in the healthcare worker cohort (all $p > 0.05$).

Comparable anti-AAV NAb prevalence between RP patients and healthy participants

In the RP group, NAb prevalence showed a similar pattern to that of the HAP group across serotypes. At a 1:50 dilution, AAV2, AAV9, and AAV2.7m8 showed prevalence rates of 72.5%, 57.5%, and 62.5%, while rate for AAV5, AAV6, and AAV8 were 27.5%, 47.5%, and 52.5% (Figure 4A). Meanwhile, a similar polarization was also observed in the RP group, with 47.5% of RP patients positive for at least five serotypes and 27.5% negative for all six serotypes (Figure 4B).

Median NAb titers were highest for AAV2 at 1:1,082 (1:622–1:3,769) and lowest for AAV5 at 1:5 (1:5–1:31) (Figure 4C). No significant differences were observed in NAb titers between the RP group and the Shanghai HAP group across all six serotypes ($p > 0.05$). No significant subgroup differences were detected based on gender and age groups (Tables S1 and S2).

High co-prevalence of NAbs among AAV2, AAV2.7m8, AAV6, and AAV8

Assessing the co-prevalence of NAb positivity across multiple AAV serotypes is essential for understanding the natural prevalence of AAV infections. We analyzed NAb co-prevalence against AAV2, AAV5, AAV6, AAV8, AAV9, and AAV2.7m8 in the HAP, healthcare workers, and RP groups. At a 1:50 dilution, the highest co-positivity was observed for NAbs against AAV2 and AAV2.7m8, reaching 65.95% in the HAP group, 73.77% in healthcare workers and 62.5% in the RP group. In contrast, AAV5 exhibited lower co-positivity with other serotypes across all groups (Figures 5A–5C).

Titer correlation analyses were consistent with these co-prevalence patterns. Among combinations involving AAV2, AAV2.7m8 showed the strongest correlation with AAV2 NAb titers ($r = 0.93$, 0.88, and 0.96 in HAP, healthcare workers, and RP groups, respectively; $p < 0.001$), followed by AAV6 ($r = 0.91$, 0.85, and 0.94; $p < 0.001$) (Figures 5D–5F). Among correlations involving AAV6 in the HAP group, the highest coefficients were observed with AAV8 ($r = 0.96$) and AAV2.7m8 ($r = 0.93$) ($p < 0.001$)

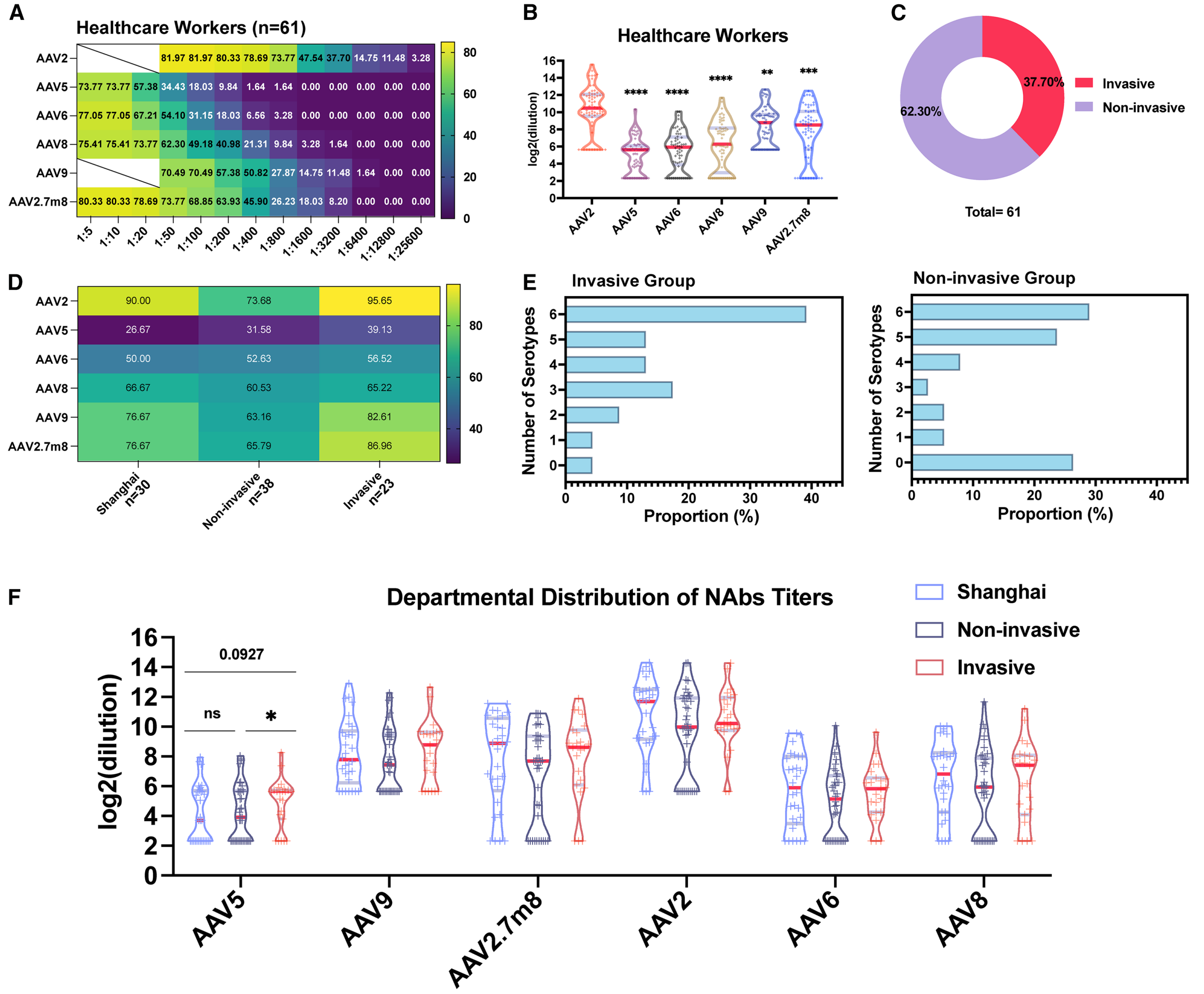

Figure 3. Prevalence and titers of anti-AAV NAbs in healthcare workers

(A) Heatmap of anti-AAV NAbs seroprevalence across different dilution levels in healthcare workers for AAV2, AAV5, AAV6, AAV8, AAV9, and AAV2.7m8. The percentage of seropositive individuals is indicated by color intensity according to the scale, with numbers inside squares representing the exact proportion at each serotype and dilution level. (B) Truncated violin plots showing the relative levels of anti-AAV NAbs titers in healthcare workers. $^{**}p < 0.01$. $^{***}p < 0.001$. $^{****}p < 0.0001$ (all compared to AAV2). (C) Proportional distribution of healthcare workers by invasiveness of procedures performed. (D) Heatmap of anti-AAV NAbs seroprevalence for AAV2, AAV5, AAV6, AAV8, AAV9, and AAV2.7m8 at 1:50 dilution in healthy adult participants from Shanghai and healthcare workers with or without invasive procedure. The percentage of seropositive individuals is indicated by color intensity according to the scale, with numbers inside squares representing the exact proportion at each serotype and dilution level. (E) Co-prevalence distribution of NAbs at 1:50 dilution in healthcare workers with or without invasive procedure. The *y* axis represents the degree of co-prevalence, ranging from negative NAb activity for all serotypes (0), partial positive for some serotypes (1–5), to positive for all serotypes (6). The *x* axis shows the proportion (%) of individuals at each co-prevalence level. (F) Truncated violin plots showing the relative levels of anti-AAV NAbs titers for AAV2, AAV5, AAV6, AAV8, AAV9, and AAV2.7m8 in healthcare workers with or without invasive procedures. $^{*}p < 0.05$. For truncated violin plots, NAb titers (ID50) are presented on a log2 scale, with transformed values for each individual displayed as scatter points overlaid on violin plots. Blue lines: quartile values; red lines: median values. Statistical comparisons between groups were performed using the Kruskal-Wallis test followed by Dunn's multiple comparisons test.

(Figure 5D). NAb titers against AAV9 consistently demonstrated their strongest correlation with AAV8, whereas NAb titers against AAV5 showed weaker correlations with other serotypes (e.g., r = 0.78 with AAV2.7m8 in HAP, r = 0.69 with AAV2 in healthcare workers; $p < 0.001$) (Figures 5D–5F).

Clustering analysis of AAV serotypes based on the titers of the six serotypes measured in each participant further visualized NAb co-prevalence patterns of NAbs. In the HAP and RP groups, AAV2 and AAV2.7m8 clustered together (Figures 5G and 5I), indicating a significant co-prevalence of NAbs against these two serotypes.

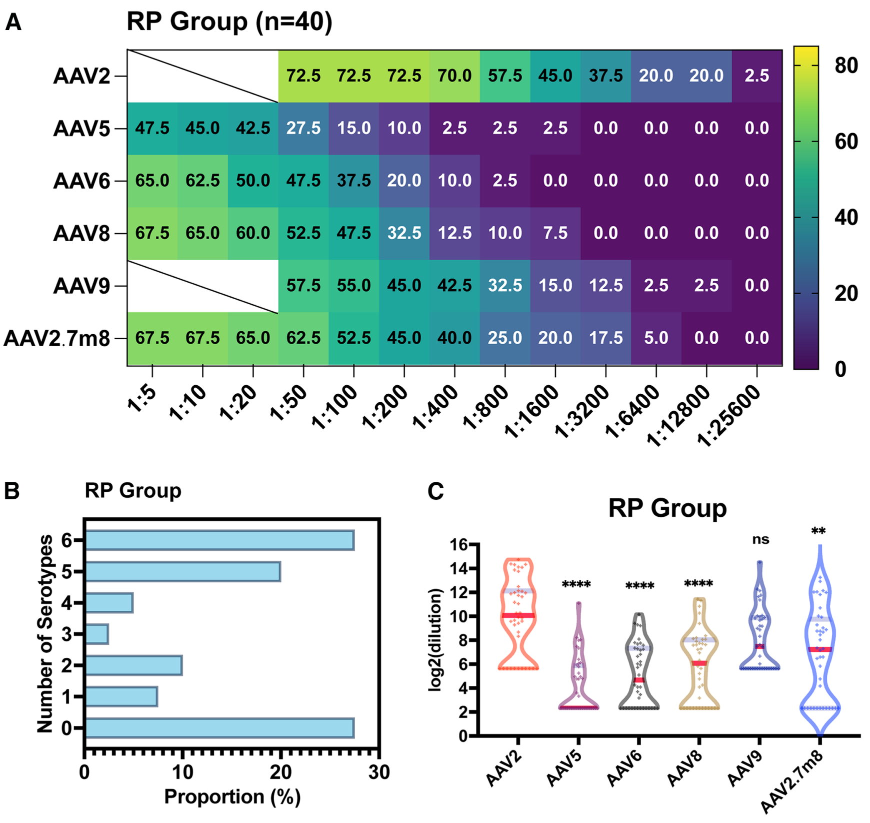

Figure 4. Prevalence and titers of anti-AAV NAbs in RP group

(A) Heatmap of anti-AAV NAbs seroprevalence across different dilution levels in RP group for AAV2, AAV5, AAV6, AAV8, AAV9, and AAV2.7m8. The percentage of seropositive individuals is indicated by color intensity according to the scale, with numbers inside squares representing the exact proportion at each serotype and dilution level. (B) Co-prevalence distribution of NAbs in RP group. The *y* axis represents the degree of co-prevalence, ranging from negative NAb activity for all serotypes (0), partial positive for some serotypes (1–5), to positive for all serotypes (6). The *x* axis shows the proportion (%) of individuals at each co-prevalence level. (C) Truncated violin plots showing the relative levels of anti-AAV NAbs titers in RP group. For truncated violin plots, NAb titers (ID50) are presented on a log2 scale, with transformed values for each individual displayed as scatter points overlaid on violin plots. Blue lines: quartile values; red lines: median values. Statistical comparisons between groups were performed using the Kruskal-Wallis test followed by Dunn's multiple comparisons test. $^{**}p < 0.01$. $^{****}p < 0.0001$. ns, no significance (all compared to AAV2).

Despite variations in subgroup clustering patterns among healthcare workers compared to the HAP and RP groups (Figure 5H), AAV2, AAV2.7m8, AAV6, and AAV8 consistently formed a major cluster, suggesting shared immunological properties or cross-reactivity. AAV5 clustered most distantly from the other five serotypes in both the HAP and RP groups (Figures 5G and 5I). Given that AAV-induced host immune responses and cellular tropism are closely associated with AAV capsid proteins (VP), we conducted a phylogenetic analysis of the VP proteins of the six AAV serotypes (Figure 5J). Results showed that the AAV5 VP protein was evolutionarily most divergent from the other serotypes, consistent with its lower NAb titer correlation and distant clustering. The evolutionary proximity of AAV2 to AAV2.7m8 and AAV9 to AAV8 partially explains their consistency in NAb titer correlations and clustering patterns.

Recursive feature elimination identifies association between anti-AAV NAb levels and hepatic lipid metabolism

We employed the tree-based LightGBM algorithm to model AAV NAb titers among healthy participants from Shanghai. Hematological and biochemical indicators were used to conduct recursive feature elimination to screen for features strongly associated with NAbs against AAV5, AAV6, AAV8, AAV9, and AAV2.7m8. Overall, for AAV2, AAV2.7m8, AAV6, and AAV8, NAb titers were associated with multiple liver function indicators, specifically those related to lipid metabolism, such as total cholesterol, triglycerides, and apolipoproteins. Secondary associations included thyroid function indicators and monocyte percentage (Figure 6A).

Thyroid-stimulating hormone (TSH) was a broadly associated feature, showing a positive relationship between SHAP (Shapley Additive explanations) values and NAb titers for all serotypes except AAV5, indicating that higher TSH levels corresponded to higher NAb titers (Figures 6B–6F).

Features in the liver function panel did not show uniform associations across all AAV serotypes. The most broadly associated features were total cholesterol and hepatitis B surface antibody (HBsAb), both exhibiting positive associations with NAb titers. Serum total cholesterol correlated positively with AAV2, AAV6, and AAV5 NAb titers (Figures 6B, 6D, and 6G). HBsAb levels were positively associated with AAV2, AAV6, and AAV2.7m8 NAb titers (Figures 6B–6D). In contrast, apolipoprotein A1 and apolipoprotein B, both involved in lipid transport, displayed inconsistent patterns: apolipoprotein A1 showed a positive association with AAV2.7m8 and AAV8 NAb titers (Figures 6C and 6E), whereas apolipoprotein B was negatively associated with AAV2 NAb titers (Figure 6B).

Among hematological features, monocyte proportion was broadly associated with NAb titers, with a negative relationship observed

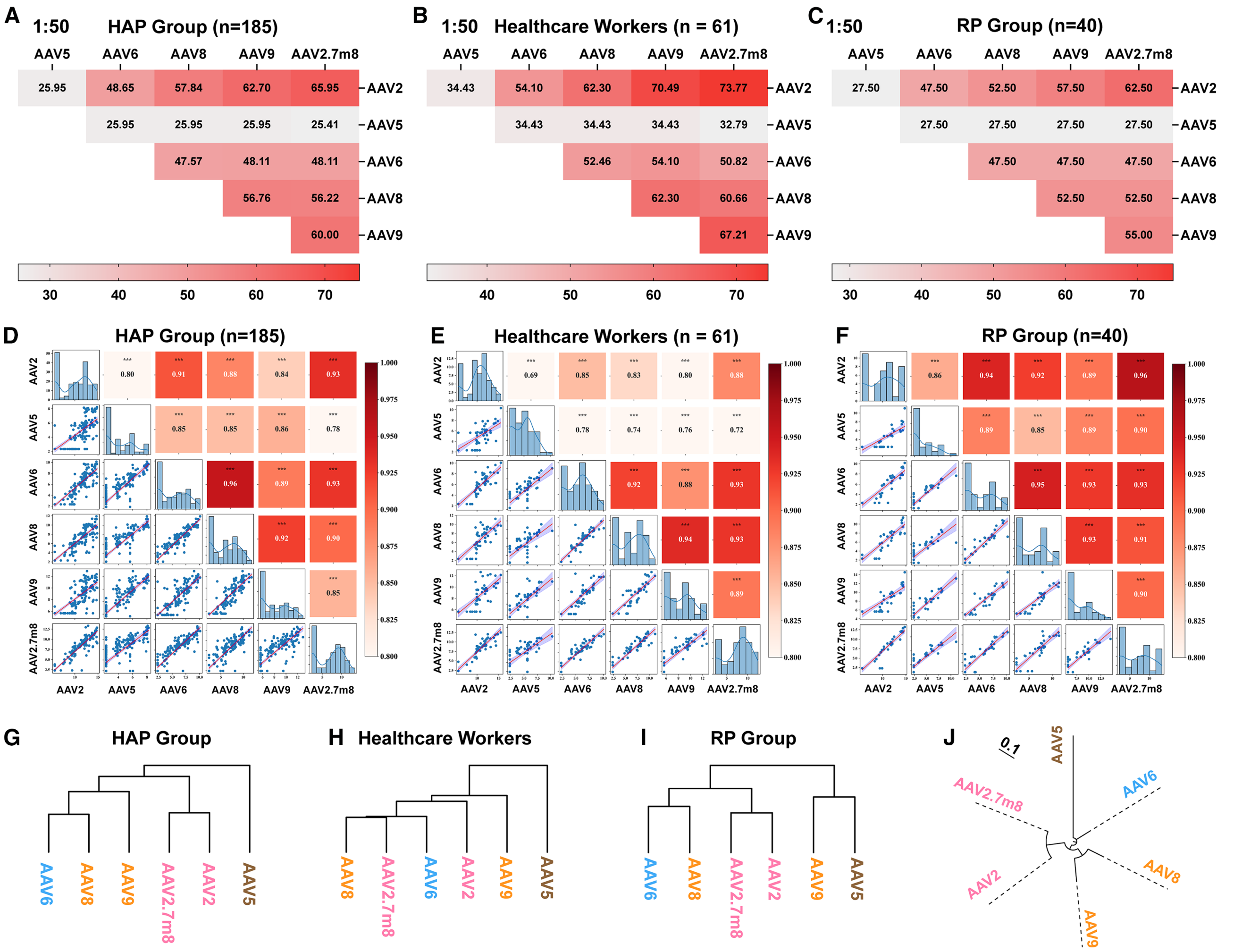

Figure 5. Correlation analysis of NAbs titers across different serotypes

(A–C) Heatmap showing the percentage of individuals with co-positive NAb activity for two serotypes at 1:50 dilution across the HAP group, healthcare workers, and RP group. Color intensity indicates the percentage of co-positive individuals according to the scale, with numbers inside squares representing the exact proportion. (D–F) Correlation coefficient heatmap (upper, right), scatterplot distribution (lower, left), and frequency distribution (diagonal) of NAbs between serotypes in HAP group, healthcare workers, and RP group. Correlation coefficients are indicated by color intensity according to the scale. ***, $p < 0.001$. Correlations were calculated using log2-transformed NAb titers (ID50). ***, $p < 0.001$. (G–I) Hierarchical clustering dendrograms of six serotypes based on log2-transformed NAb titers (ID50) across all individuals. Serotypes are clustered according to their NAb titer patterns across individuals, with serotypes within the same cluster exhibiting concordant NAb titer trends. (J) Phylogenetic tree constructed based on VP1 protein sequences of different serotypes. Protein sequences under same node are marked with the same color. Serotype colors match (G–I).

between monocyte proportion and NAb titers for AAV2, AAV2.7m8, AAV8, and AAV9, indicating that lower monocyte proportions tend to correlate with higher NAb titers (Figures 6C and 6E–6F).

DISCUSSION

Pre-existing NAbs against AAVs can significantly impede vector delivery and target cell transduction in AAV-mediated gene therapies. Determining NAb titers for specific AAV serotypes is therefore critical for evaluating therapeutic efficacy and guiding patient selection in both preclinical and clinical settings. Despite the expanding use of AAV-based gene therapies, detailed population-level measurement of pre-existing NAbs, especially in the Chinese population, remains limited.

This study reports the NAb prevalence and titers for AAV2, AAV5, AAV6, AAV8, AAV9, and AAV2.7m8 across healthy populations from six regions in northern and southern China, as well as in patients with RP and healthcare workers in Shanghai. These data provide valuable insights for designing clinical trials and screening cutoffs in NAbs for gene therapy applications. Among the six serotypes analyzed, AAV2 exhibited the highest NAb seroprevalence and titer

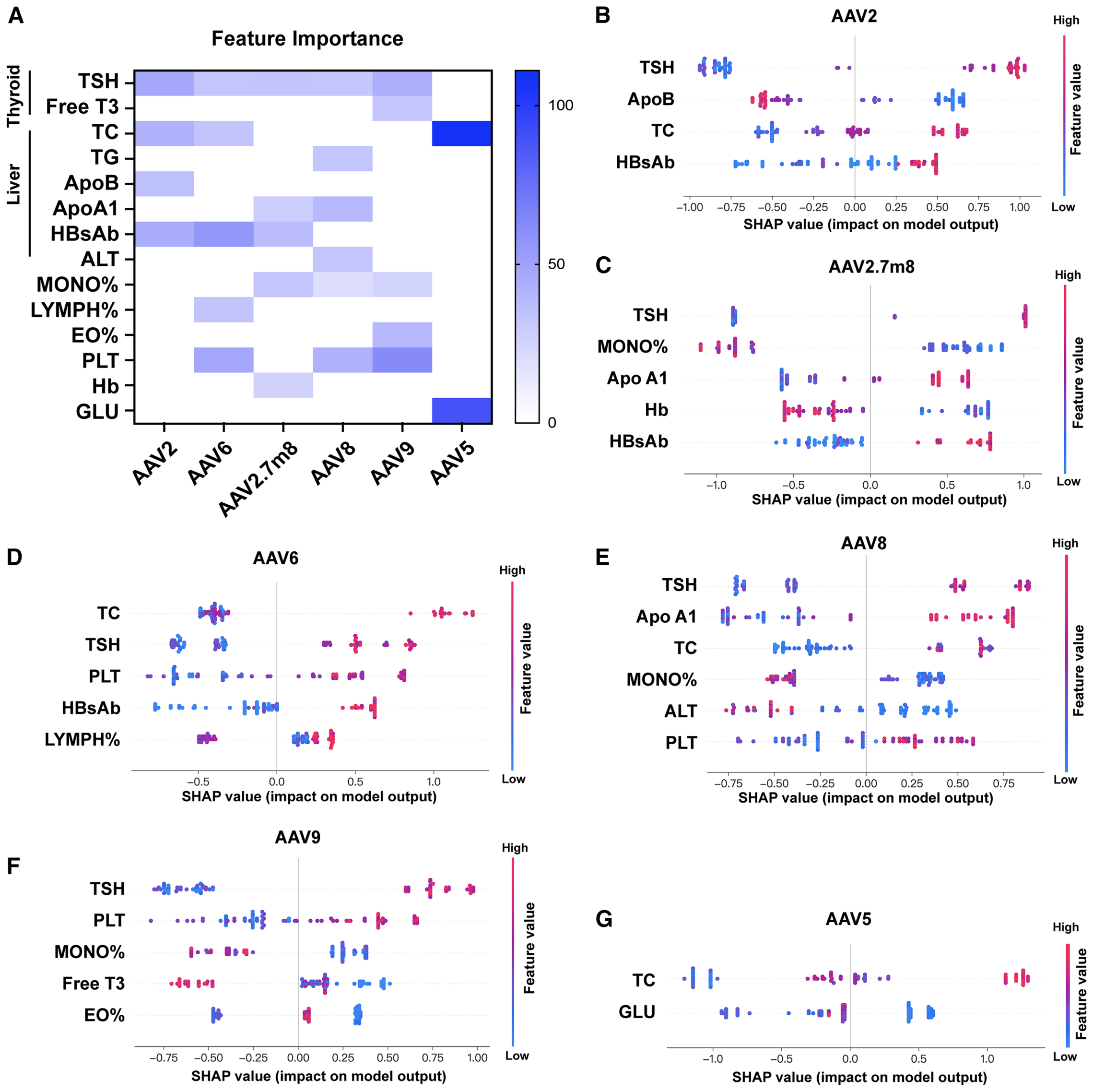

Figure 6. Machine learning-based identification of physiological features associated with anti-AAV NAb titers

(A) Feature-importance heatmap of the top predictive physiological features for NAb titers. Important values indicated by color intensity according to scale. Feature importance reflects the strength of association between each physiological factor and NAb titer levels as determined by the machine learning model, with higher values indicating stronger predictive associations. (B–G) SHAP plots illustrating the marginal effects of representative physiological features on predicting NAb titers. Each dot represents one participant; the color gradient (blue to red) denotes the feature value (low to high). For a given feature, high-value (red) points concentrated on the positive side reflect a positive association between higher feature values and higher NAb titers; high-value points concentrated on the negative side reflect a negative association. TSH, thyroid-stimulating hormone; free T3, free triiodothyronine; TC, total cholesterol; TG, triglycerides; ApoB, apolipoprotein B; ApoA1, apolipoprotein A1; HBsAb, hepatitis B surface antibody; ALT, alanine aminotransferase; MONO%, monocyte percentage; LYMPH%, lymphocyte percentage; EO%, eosinophil percentage; PLT, platelet count; Hb, hemoglobin; GLU, plasma glucose.

across all groups (Figure 1), consistent with findings from previous research.[15] The high prevalence of NAbs against AAV2 is potentially related to both its efficient binding ability to various cell surface receptors, such as heparan sulfate proteoglycans, and widespread early life exposure to this serotype.[17] AAV5 exhibited the lowest NAb positivity and titer, potentially attributed to its restricted receptor distribution (e.g., sialic acid-associated receptors) and low natural infection rate, which limit immune exposure.[18]

Previous studies have reported geographic and racial differences in the prevalence of anti-AAV NAbs, with Asian populations exhibiting higher NAb levels compared to African and Caucasian populations.[8]

We further reviewed existing studies on AAV seroprevalence in China, and our results generally align with previous findings. Consistent with prior reports, AAV2 exhibited the highest seroprevalence. In our HAP cohort, the AAV2 seroprevalence was 72.43% at 1:50 dilution, which is lower than 89.4% reported by Liu et al. from two Chinese provinces and the 97.4% rate reported by Qin et al. in normal subjects.[15,16] Similarly, we observed a relatively lower seroprevalence for AAV5, consistent with previous reports. In our HAP cohort, the AAV5 seroprevalence was 57.3% at 1:10 dilution, which is slightly higher than 40.2% reported by Liu et al. in normal subjects, and exceeds the rates observed in other Asian populations, such as Japan (22%) and South Korea (36%).[8,15] Such variability across studies aligns with the observations that anti-AAV NAb seroprevalence exhibits significant geographic heterogeneity.[8,11,16] In this study, unsupervised clustering and statistical analyses revealed a trend of higher NAb titers in southern Chinese populations compared to northern populations (Figures 2A and 2B; Table 2), particularly for AAV6 and AAV8. Environmental factors, including temperature, humidity, population density, and lifestyle differences, may be associated with the observed variations in NAb titer patterns between northern and southern regions.[19]

Among participants from Shanghai, healthcare workers performing frequent invasive procedures exhibited higher anti-AAV NAb titers compared to staff performing non-invasive procedures (Figures 3D–3F; Table 2). Previous studies have found that occupations involving frequent invasive procedures are associated with higher exposure to blood and body fluids.[20] These occupational characteristics increase exposure to potentially infectious biological samples, given that hepatitis B, hepatitis C, and cytomegalovirus (CMV) commonly transmit through this route.[21] Furthermore, certain helper viruses, such as CMV, can synergistically enhance AAV infection.[22] However, whether the higher NAb titers (particularly against AAV5) observed in healthcare workers performing invasive procedures are associated with potential exposure to body fluids, and whether AAV can transmit among humans via infectious body fluids or excreta, or whether helper viruses facilitate variations in immune recognition and NAb responses, warrants further investigation.

The analysis of AAV NAb co-prevalence provides insights into the natural infection patterns and transmission modes of AAV, while also aiding in the evaluation of whether different serotypes may be subject to similar humoral immune suppression. Our study revealed a significant co-prevalence of NAbs against AAV2 and AAV2.7m8, reflecting their close phylogenetic relationship (Figures 5A–5C). AAV2.7m8 is derived from AAV2 with a nine-amino-acid insertion at VP position 587, enhancing its tropism.[23] Their shared epitopes likely contribute to overlapping NAb responses, with high co-prevalence of NAbs due to conserved sequences. Notably, the insertion of a short peptide into AAV2 to generate AAV2.7m8 resulted in reduced anti-AAV2.7m8 NAb titers and lower prevalence at the same dilution (Figures 1A and 1B), suggesting a new strategy for developing immune-evading vectors. By modifying and screening the *cap* gene to minimize recognition of conserved regions, it is possible to design AAV variants with enhanced immune evasion and improved cellular infection efficiency. AAV8 and AAV9, which also share close evolutionary distances in their VP proteins, demonstrated strong associations in titer correlation (Figures 5D–5F). In contrast, AAV5, with a more distant evolutionary relationship, exhibited lower co-prevalence. These findings underscore the influence of capsid sequence homology on humoral immunity and vector design.

Compared to simple linear regression, machine learning regression models demonstrate superior robustness and generalizability, enabling more reliable feature mining.[24] Our analysis identified statistical associations between AAV NAb titers and lipid metabolism parameters, including cholesterol levels, triglycerides, and apolipoproteins. Previous studies have reported that pre-existing NAbs could arise from prior exposure to wild-type AAVs, which induce adaptive immune responses.[2] Most AAV serotypes exhibit strong natural hepatotropism after entering circulation.[25] Although systematic clinical evidence linking AAV liver accumulation to altered hepatic function is limited, recent reports, including studies published in *Nature*, have reported multiple cases linking AAV2 infection to an unexplained surge in acute hepatitis among children, though the virus's role in liver disease development remains unclear.[26–28] These existing lines of evidence suggest a potential association between AAV infection and hepatic function. Given that the liver is the primary organ for cholesterol and triglyceride metabolism, the specific biological significance underlying the observed associations between NAb titers and these lipid parameters, and whether these associations relate to subclinical liver alterations, warrants further experimental investigation.[29] Notably, variables such as pathogen exposure intensity and the frequency, or complexity of patient contact were not accounted for in the current model. Given the observation that staff involved in invasive procedures presented with higher median AAV5 NAb titers among healthcare workers, mining the association between pathogen exposure intensity and NAb seroprevalence could yield valuable insights for future research.

Clinically, rAAV-mediated gene therapy, as an interventional approach, has provided more direct evidence of hepatic injury, challenging the traditional belief that AAV is benign. rAAV-based therapy was reported to induce transient transaminase elevations, and in rare cases, severe hepatic injury.[30,31] For instance, valoctocogene roxaparvovec (AAV-hFVIII-SQ) administration led to severe alanine aminotransferase (ALT) elevations in two patients who subsequently required intravenous (i.v.) methylprednisolone.[32] More critically, at the time of writing, Sarepta Therapeutics' Elevidys (rAAVrh74) for Duchenne muscular dystrophy (DMD) has resulted in three deaths due to acute liver failure.[33] One current theory proposes that innate immune responses to AAV lead to high levels of NAbs and virus-antibody complexes that trigger complement-mediated hepatic inflammation, while another suggests co-infection with viruses such as HAdV ultimately causes hepatocellular damage and carcinogenesis.[26,34,35] Given the potential role of pre-existing NAbs in such immune-mediated injury, screening for anti-AAV NAb titers during

gene therapy candidate selection becomes particularly important, along with exploring strategies to reduce NAb titers prior to treatment.

This study has several limitations. Our findings should be interpreted as providing insights into geographic distribution patterns rather than definitive national estimates, as we employed convenience sampling from selected provinces with limited sample sizes per region, which may not fully represent the general Chinese population. Besides, our conclusions cannot be extended to pediatric populations. Our findings from 40 single-center RP patients may not represent the broader IRD (inherited retinal diseases) population, highlighting the need for future studies with diverse IRD subtypes. While our study identified significant associations between lipid profiles [TC (total cholesterol), HDL (high-density lipoprotein), and LDL (low-density lipoprotein)] and NAb titers within a Chinese population, the generalizability of these findings across different ethnic groups remains to be verified in future multi-ethnic cohort studies.

There are potential sources of statistical bias in our analysis. Although the primary north-south comparison of NAb titers was not confounded by age or gender distributions, the assessment of these variables as confounders at the province-level was limited by low events per variable (EPV) ratios, potentially compromising the stability of parameter estimates.[36] Accordingly, the unadjusted differences in NAb seroprevalence and titers stratified by province, gender, or age group are presented as exploratory findings, since the difference observed in the unadjusted comparisons may be attributable to heterogeneity in demographic variables across provinces, such as the significant imbalances in age and gender distributions, or to other unknown or unmeasured demographic factors. Future studies with larger sample sizes, allowing for the adjustment of multiple potential confounders, will facilitate the identification of more robust factors associated with NAb seroprevalence. Furthermore, given the number of comparisons performed, marginal *p* values should be interpreted with caution. Testing for AAV2 and AAV9 was initiated at a 1:50 dilution due to expected high seroprevalence, which may affect sensitivity and precision at lower dilutions. While this approach facilitated the quantification of high-titer samples, it may have excluded participants with very low NAb levels. This should be considered when comparing seroprevalence rates across specific serotypes.

Conclusion

This study provides a comprehensive characterization of anti-AAV NAb prevalence and associated physiological features across six Chinese provinces. AAV2 exhibited the highest seroprevalence and NAb titers, while AAV5 showed the lowest. Southern provinces showed higher seroprevalence of anti-AAV6 and anti-AAV8 NAbs than northern provinces. Notably, samples from Shanghai and Harbin exhibited higher NAb titers across multiple serotypes than other regions. Healthcare workers with invasive procedures demonstrated higher NAb prevalence and titers. Co-prevalence analysis revealed strong associations between AAV2 and AAV2.7m8, as well as AAV8 and AAV9, whereas AAV5 showed minimal co-prevalence, consistent with the evolutionary divergence in their capsid VP sequences. Furthermore, machine learning-based analysis further identified statistical associations between NAb titers and lipid profiles (TC, HDL, and LDL). Given that the liver is the primary organ for lipid metabolism and the hepatotropism for both wild-type AAV and recombinant AAV vectors, the biological mechanisms underlying these associations and their potential relationship to liver function warrant further experimental investigation.

MATERIALS AND METHODS

Participants

This multi-provincial, cross-sectional, epidemiologic study included participants across six Chinese provinces (Beijing, Shandong, Harbin, Hunan, Guangdong, and Shanghai). HAP were recruited based on the following inclusion criteria: age between 18 and 60 years, subjects and legal guardians' informed consent to additional laboratory examinations, and normal routine examinations (including complete blood count, liver and kidney function tests, blood glucose, chest radiography, and abdominal ultrasound). Exclusion criteria encompassed: glucocorticoid or immunosuppressant use within 1-year, major surgery or trauma within 6 months, previous gene therapy exposure, systemic diseases, acute infections (such as pharyngitis and pneumonia), and blood-borne pathogen infections (including HBV, HCV, and HIV). Healthcare workers meeting the HAP inclusion and exclusion criteria were further stratified into invasive and non-invasive groups based on whether they performed invasive procedures. An invasive procedure was defined as one involving purposeful access to the body through incision, percutaneous puncture with instrumentation beyond the puncture needle, or instrumentation via natural orifices, beginning when body entry is gained and ending when instruments are removed or skin is closed, including but not limited to the use of endoscopes, catheters, scalpels, scissors, devices, and tubes.[37] Patients aged up to 60 years with the diagnosis of RP were consecutively recruited at Shanghai General Hospital. The diagnosis decisions were confirmed by at least two of the experienced specialists (X.S., T.L., and J.S.) for each patient based on clinical phenotype, family history, electrophysiologic testing, and genetic testing. Basic characteristics including age, sex, residence, medication history, surgical, and trauma history, were collected from all participants. The study enrolled 246 HAPs (including 61 healthcare workers) and 40 RP patients, with demographic data presented in Table 1.

Study design

The primary objective was to evaluate the serological prevalence of NAbs against gene therapy-related AAV serotypes (AAV2, AAV5, AAV6, AAV8, AAV9, and AAV2.7m8) among participants. Secondary objectives included: (1) differential analysis of NAbs serological prevalence among HAP and Shanghai RP cohorts; (2) assessment of NAb serological prevalence variations across healthcare workers in Shanghai; (3) evaluation of NAb co-prevalence across serotypes in HAP, healthcare workers, and RP groups. The exploratory

objective involved developing prediction models using the LightGBM algorithm to identify key physiological features associated with NAb titers across serotypes.

Validated NAb assay

The TI assay for each AAV serotype was validated in accordance with the FDA guidance of developing and validating assays for anti-drug antibody detection and relevant white papers.[38–41] The validation parameters included intra- and inter-assay precision, selectivity, drug tolerance, and stability. All validation results met the pre-established acceptance criteria. Following informed consent, 5 mL whole blood sample was collected via venipuncture using vacuum tubes and 22G needles, settled at room temperature for 30 min, and centrifuged at 1,500× g for 10 min. Serum was aliquoted (500 μL/tube), flash-frozen in liquid nitrogen, and stored at −80°C until analysis. NAbs against AAV2, AAV5, AAV6, AAV8, AAV9, and AAV2.7m8 were detected using validated TI assay (Tables S3–S8).[2] Briefly, AAV-CMV-luciferase vectors packaged with capsids of different serotypes were constructed and purified by cesium chloride ultracentrifugation for HEK293T cell infection. Serotype-specific NAbs in serum samples can specifically bind to corresponding AAV vectors, inhibiting their transduction and resulting in reduced luciferase expression. After thawing, serum samples were serially diluted (1:5 to 1:25,600). For AAV2 and AAV9, dilutions started at 1:50 to optimize sensitivity and precision given their high baseline seroprevalence. The diluted sera were then mixed with the corresponding AAV-CMV-luciferase vectors and incubated at room temperature (600 rpm, 1 h). The multiplicity of infection (MOI) used for each AAV serotype and the baseline relative luminescence are listed in Table S9.

Titer negative control was generated by combining serum from individuals showing luciferase signals comparable to baseline cell culture media, confirming minimal to no AAV neutralization capacity. Recombinant mouse anti-AAV antibodies (Progen) were used, including anti-AAV2 (A20R, Cat. No. 610298, lot no. 810021–05 for AAV2; lot no. FAK21048-21 for AAV2.7m8), anti-AAV5 (ADK5b, Cat. No. 610149, lot no. 608021–09), anti-AAV6 (ADK6, Cat. No. 690159, lot no. FAK23204-01), anti-AAV8 (ADK8, Cat. No. 610160, lot no. 608101–08), and anti-AAV9 (ADK9, Cat. No. 690162, lot no. FAK21007-10), to prepare positive controls by spiking antibodies at specified concentrations (Table S3–S8) into the pooled negative control serum to generate titer positive controls, which were incubated with AAV vectors following the same test protocol as serum samples. The mixtures were then added to HEK293T cells seeded in 96-well plates and incubated at 37°C, 5% CO2 for 24 h. After incubation, 60 μL/well of Bio-LiteTM luciferase substrate (Vazyme, Cat. No. DD1201) was added and incubated in darkness for 20 min. Luminescence signals were measured using Spectra Max L (Luminescence mode, Integration: 500 ms), with signal intensity inversely proportional to NAbs activity.

Seroprevalence and NAb titers

In the TI-based assay, NAb titers were reported as ID50, defined as the serum dilution that induced 50% inhibition of the luciferase signal relative to the negative control, calculated by four-parameter curve fitting (interpolation) across all tested dilutions.[2] Seroprevalence was assessed based on qualitative NAb determinations at each serum dilution (positive if ID50 > the dilution, negative if ID50 < the dilution).[8] Seroprevalence was assessed based on qualitative NAb determinations (positive, negative) at each serum dilution across serotypes.

Machine learning analysis

For regional and department clustering, statistical features of NAb titers (mean, median, SD, 10th percentile, 25th percentile, 75th percentile, 90th percentile, median absolute deviation, skewness, kurtosis, max/median, and 75th/25th percentile ratio) were extracted and analyzed using hierarchical clustering (method = ward. D2) in the cluster package. AAV NAb co-prevalence analysis employed hierarchical clustering (method = ward. D2) based on NAb titers from all participants, with optimal cluster numbers determined by silhouette coefficients.

Sequence comparison was performed using IQ Tree software for phylogenetic inference, constructing topological phylogenetic trees (Bootstrap = 2,000) for VP1 sequences of AAV2 (P03135), AAV5 (Q9YIJ1), AAV6 (O56137), AAV8 (Q8JQF8), AAV9 (Q6JC40), and AAV2.7m8 (LALGETTRP sequence inserted after amino acid position 587 in AAV2 VP1 protein).[42]

Light Gradient Boosting Machine (LightGBM) is a gradient boosting framework that implements tree-based learning algorithms optimized for handling large-scale data with high efficiency and accuracy.[43] In this study, the LightGBM model was employed to identify features associated with NAb titers. Data from HAPs and healthcare workers from Shanghai were used for feature mining. For data preprocessing, features including demographics, hematology, liver and renal function panels, glucose, lipid metabolism, thyroid function, and immunological indicators, as well as the clinical working years of healthcare workers, were collected. Pearson's correlation analysis was performed to identify highly correlated features, and for feature pairs with absolute correlation coefficients greater than 0.3, the more representative feature was retained to reduce multicollinearity. Categorical variables were transformed based on one-hot encoding. After preprocessing, 44 features were included for model input. The dataset was then split into training (80%) and test (20%) sets. All LightGBM models were trained with the following hyperparameters: regression objective, 31 leaves per tree, learning rate of 0.05, 100 estimators, and fixed random seed of 42. Model performance was evaluated using 5-fold cross-validation on the training set, with root mean square error (RMSE) and R-squared (R^2) calculated across folds to guide feature selection. Recursive feature elimination with cross-validation (RFECV) method was applied to iteratively remove the least important feature based on LightGBM feature importance scores until the optimal feature subset with minimum cross-validated RMSE was identified. Final model performance was assessed on the held-out test set. To quantify the contribution of each retained key feature to model predictions, SHAP values were computed using

TreeExplorer and visualized using global summary (beeswarm) plots. The pseudo-code for the entire feature selection and model training pipeline is provided in supplemental material.

Statistical analysis

All statistical analyses were performed using GraphPad Prism 10, R 4.3.1, and Python 3.11.2. Categorical variables, including gender, geographical region, and grouping status, were described as frequencies or percentages. Due to the exponential distribution of NAbs dilutions, titers were log2-transformed to stabilize variance and approximate normal distribution, and were presented as median with 95% confidence intervals. Multivariable logistic regression models were constructed to assess the respective contributions of age, sex, and geographic region (north vs. south) to NAb titers. NAb titers were dichotomized at the median to define the binary outcome. VIFs were calculated to evaluate multicollinearity among covariates. Kolmogorov-Smirnov test was used to assess normality of continuous variables. Mann-Whitney *U* test was applied to compare values between two groups. For comparisons involving >3 groups, Kruskal-Wallis test followed by post hoc multiple comparison tests were performed. Correlation coefficients were measured using Spearman's correlation. Statistical significance was defined as $p < 0.05$.

DATA AND CODE AVAILABILITY

The study data and supplemental code required for reanalysis are available from the corresponding author upon request.

ACKNOWLEDGMENTS

This work was supported by the National Natural Science Foundation of China (82571246, 82388101, and U22A20311), and Science and Technology Commission of Shanghai Municipality (23J41900200), and Shanghai "Rising Stars of Medical Talents" Youth Development Program (SHWSRS(2025)_071). This study was approved by the Institutional Review Board ([2025]138) of Shanghai General Hospital and was conducted in accordance with the Declaration of Helsinki. All participants provided written informed consent before recruitment. Local community volunteers were actively involved in the recruitment of this study. Patients or the public were not involved in the design, conduct, reporting, or dissemination plans of the research.

AUTHOR CONTRIBUTIONS

J.D., H.J., X.X., T.L., and X.S. conceived and designed the study; Z.L., H.Z., Z.L., W.G., and X.T. carried out laboratory experiments and data acquisition; X.L., T.Z., J.C., and J.S. performed preliminary data processing and initial analyses; Y.W., Z.L., H.Z., and H.J. coordinated and oversaw regional surveys and sample collection; J.D. and T.L. supervised laboratory experiments and contributed to data interpretation; T.L., X.S., and H.J. verified the underlying data; J.D. and T.L. conducted statistical analyses, prepared the figures, and drafted the manuscript; T.L., X.X., H.J., and X.S. critically reviewed and revised the manuscript. All authors had full access to all data, approved the final version, and accept responsibility for the decision to submit for publication.

DECLARATION OF INTERESTS

X.S. reported being consultants of Novartis, Roche, Alcon, Allergan, Bayer Healthcare, Innovent Biologics Inc., Kanghong Biotech Inc., and Carl Zeiss Meditec Inc. T.L. reported being consultant of Innostellar Biotherapeutics Co., Ltd. Z.L., W.G., and X.T. are empolyees of Shanghai Xihua Scientific Co., Ltd. T.L. reported receiving grants from the National Nature Science Foundation of China. X.S. reported receiving grants from the National Key R&D Program of China and the National Nature Science Foundation of China. No other disclosures were reported.

SUPPLEMENTAL INFORMATION

Supplemental information can be found online at https://doi.org/10.1016/j.omta.2026.201743.

REFERENCES

1. Wang, J.H., Gessler, D.J., Zhan, W., Gallagher, T.L., and Gao, G. (2024). Adeno-associated virus as a delivery vector for gene therapy of human diseases. Signal Transduct. Targeted Ther. *9*, 78.
2. Schulz, M., Levy, D.I., Petropoulos, C.J., Bashirians, G., Winburn, I., Mahn, M., Somanathan, S., Cheng, S.H., and Byrne, B.J. (2023). Binding and neutralizing anti-AAV antibodies: Detection and implications for rAAV-mediated gene therapy. Mol. Ther. *31*, 616–630.
3. Mingozzi, F., and High, K.A. (2013). Immune responses to AAV vectors: overcoming barriers to successful gene therapy. Blood *122*, 23–36.
4. Wang, L., Calcedo, R., Bell, P., Lin, J., Grant, R.L., Siegel, D.L., and Wilson, J.M. (2011). Impact of pre-existing immunity on gene transfer to nonhuman primate liver with adeno-associated virus 8 vectors. Hum. Gene Ther. *22*, 1389–1401.
5. Manno, C.S., Pierce, G.F., Arruda, V.R., Glader, B., Ragni, M., Rasko, J.J., Ozelo, M.C., Hoots, K., Blatt, P., Konkle, B., et al. (2006). Successful transduction of liver in hemophilia by AAV-Factor IX and limitations imposed by the host immune response. Nat. Med. *12*, 342–347.
6. Shen, W., Liu, S., and Ou, L. (2022). rAAV immunogenicity, toxicity, and durability in 255 clinical trials: A meta-analysis. Front. Immunol. *13*, 1001263.
7. Liu, Z., Zhang, H., Jia, H., Wang, H., Huang, Z., Tang, Y., Wang, Z., Hu, J., Zhao, X., Li, T., and Sun, X. (2025). The clinical safety landscape for ocular AAV gene therapies: A systematic review and meta-analysis. iScience *28*, 112265.
8. Chhabra, A., Bashirians, G., Petropoulos, C.J., Wrin, T., Paliwal, Y., Henstock, P.V., Somanathan, S., da Fonseca Pereira, C., Winburn, I., and Rasko, J.E.J. (2024). Global seroprevalence of neutralizing antibodies against adeno-associated virus serotypes used for human gene therapies. Mol. Ther. Methods Clin. Dev. *32*, 101273.
9. George, L.A., Sullivan, S.K., Giermasz, A., Rasko, J.E.J., Samelson-Jones, B.J., Ducore, J., Cuker, A., Sullivan, L.M., Majumdar, S., Teitel, J., et al. (2017). Hemophilia B Gene Therapy with a High-Specific-Activity Factor IX Variant. N. Engl. J. Med. *377*, 2215–2227.
10. Majowicz, A., Nijmeijer, B., Lampen, M.H., Spronck, L., de Haan, M., Petry, H., van Deventer, S.J., Meyer, C., Tangelder, M., and Ferreira, V. (2019). Therapeutic hFIX Activity Achieved after Single AAV5-hFIX Treatment in Hemophilia B Patients and NHPs with Pre-existing Anti-AAV5 NABs. Mol. Ther. Methods Clin. Dev. *14*, 27–36.
11. Calcedo, R., Vandenberghe, L.H., Gao, G., Lin, J., and Wilson, J.M. (2009). Worldwide epidemiology of neutralizing antibodies to adeno-associated viruses. J. Infect. Dis. *199*, 381–390.
12. Meliani, A., Leborgne, C., Triffault, S., Jeanson-Leh, L., Veron, P., and Mingozzi, F. (2015). Determination of anti-adeno-associated virus vector neutralizing antibody titer with an in vitro reporter system. Hum. Gene Ther. Methods *26*, 45–53.
13. Gorovits, B., Fiscella, M., Havert, M., Koren, E., Long, B., Milton, M., and Purushothama, S. (2020). Recommendations for the Development of Cell-Based Anti-Viral Vector Neutralizing Antibody Assays. AAPS J. *22*, 24.
14. Jungmann, A., Müller, O., and Rapti, K. (2017). Cell-Based Measurement of Neutralizing Antibodies Against Adeno-Associated Virus (AAV). Methods Mol. Biol. *1521*, 109–126.
15. Liu, Q., Huang, W., Zhang, H., Wang, Y., Zhao, J., Song, A., Xie, H., Zhao, C., Gao, D., and Wang, Y. (2014). Neutralizing antibodies against AAV2, AAV5 and AAV8 in healthy and HIV-1-infected subjects in China: implications for gene therapy using AAV vectors. Gene Ther. *21*, 732–738.
16. Qin, X., Li, H., Zhao, H., Xiang, K., Liu, S., Lou, R., Liu, P., Dai, Y., Wang, C., and Zhang, S. (2024). Prevalence of Neutralizing Antibodies Against AAV Serotypes 2 and 9 in Healthy Participants from Multiple Centers Across China and Patients with DMD/BMD. Hum. Gene Ther. *35*, 969–977.
17. Calcedo, R., Morizono, H., Wang, L., McCarter, R., He, J., Jones, D., Batshaw, M.L., and Wilson, J.M. (2011). Adeno-associated virus antibody profiles in newborns, children, and adolescents. Clin. Vaccine Immunol. *18*, 1586–1588.

18. Seiler, M.P., Miller, A.D., Zabner, J., and Halbert, C.L. (2006). Adeno-associated virus types 5 and 6 use distinct receptors for cell entry. Hum. Gene Ther. *17*, 10–19.

19. Neira, M., Erguler, K., Ahmady-Birgani, H., Al-Hmoud, N.D., Fears, R., Gogos, C., Hobbhahn, N., Koliou, M., Kostrikis, L.G., Lelieveld, J., et al. (2023). Climate change and human health in the Eastern Mediterranean and Middle East: Literature review, research priorities and policy suggestions. Environ. Res. *216*, 114537.

20. Ji, Y., Huang, J., Jiang, G., Liu, Q., Xiao, D., and Deng, J. (2022). Investigation of the occupational exposure to blood-borne pathogens of staff at a third-class specialist hospital in 2015-2018: a retrospective study. Sci. Rep. *12*, 1498.

21. Hosoglu, S., Akalin, S., Sunbul, M., Otkun, M., and Ozturk, R.; Occupational Infections Study Group (2009). Predictive factors for occupational bloodborne exposure in Turkish hospitals. Am. J. Infect. Control *37*, 65–69.

22. McPherson, R.A., Rosenthal, L.J., and Rose, J.A. (1985). Human cytomegalovirus completely helps adeno-associated virus replication. Virology *147*, 217–222.

23. Dalkara, D., Byrne, L.C., Klimczak, R.R., Visel, M., Yin, L., Merigan, W.H., Flannery, J.G., and Schaffer, D.V. (2013). In vivo-directed evolution of a new adeno-associated virus for therapeutic outer retinal gene delivery from the vitreous. Sci. Transl. Med. *5*, 189ra76.

24. Barbiero, P., Squillero, G., and Tonda, A.P. (2020). Modeling Generalization in Machine Learning: A Methodological and Computational Study. Preprint at arXiv. https://doi.org/10.48550/arXiv.2006.15680.

25. Gao, G., Vandenberghe, L.H., Alvira, M.R., Lu, Y., Calcedo, R., Zhou, X., and Wilson, J.M. (2004). Clades of Adeno-associated viruses are widely disseminated in human tissues. J. Virol. *78*, 6381–6388.

26. Servellita, V., Sotomayor Gonzalez, A., Lamson, D.M., Foresythe, A., Huh, H.J., Bazinet, A.L., Bergman, N.H., Bull, R.L., Garcia, K.Y., Goodrich, J.S., et al. (2023). Adeno-associated virus type 2 in US children with acute severe hepatitis. Nature *617*, 574–580.

27. Morfopoulou, S., Buddle, S., Torres Montaguth, O.E., Atkinson, L., Guerra-Assunção, J.A., Moradi Marjaneh, M., Zennezini Chiozzi, R., Storey, N., Campos, L., Hutchinson, J.C., et al. (2023). Genomic investigations of unexplained acute hepatitis in children. Nature *617*, 564–573.

28. Ho, A., Orton, R., Tayler, R., Asamaphan, P., Herder, V., Davis, C., Tong, L., Smollett, K., Manali, M., Allan, J., et al. (2023). Adeno-associated virus 2 infection in children with non-A-E hepatitis. Nature *617*, 555–563.

29. Alves-Bezerra, M., and Cohen, D.E. (2017). Triglyceride Metabolism in the Liver. Compr. Physiol. *8*, 1–8.

30. Mücke, M.M., Fong, S., Foster, G.R., Lillicrap, D., Miesbach, W., and Zeuzem, S. (2024). Adeno-associated viruses for gene therapy - clinical implications and liver-related complications, a guide for hepatologists. J. Hepatol. *80*, 352–361.

31. Larrey, D., Delire, B., Meunier, L., Zahhaf, A., De Martin, E., and Horsmans, Y. (2024). Drug-induced liver injury related to gene therapy: A new challenge to be managed. Liver Int. *44*, 3121–3137.

32. Ozelo, M.C., Mahlangu, J., Pasi, K.J., Giermasz, A., Leavitt, A.D., Laffan, M., Symington, E., Quon, D.V., Wang, J.D., Peerlinck, K., et al. (2022). Valoctocogene Roxaparvovec Gene Therapy for Hemophilia A. N. Engl. J. Med. *386*, 1013–1025.

33. FDA Safety Communication (2025). FDA Investigating Deaths Due to Acute Liver Failure Following Treatment with Sarepta's AAVrh74 Gene Therapies. https://www.fda.gov/vaccines-blood-biologics/safety-availability-biologics/fda-investigating-deaths-due-acute-liver-failure-following-treatment-sareptas-aavrh74-gene-therapies;.

34. Ertl, H.C.J. (2022). Immunogenicity and toxicity of AAV gene therapy. Front. Immunol. *13*, 975803.

35. Nault, J.C., Datta, S., Imbeaud, S., Franconi, A., Mallet, M., Couchy, G., Letouzé, E., Pilati, C., Verret, B., Blanc, J.F., et al. (2015). Recurrent AAV2-related insertional mutagenesis in human hepatocellular carcinomas. Nat. Genet. *47*, 1187–1193.

36. Peduzzi, P., Concato, J., Kemper, E., Holford, T.R., and Feinstein, A.R. (1996). A simulation study of the number of events per variable in logistic regression analysis. J. Clin. Epidemiol. *49*, 1373–1379.

37. Cousins, S., Blencowe, N.S., and Blazeby, J.M. (2019). What is an invasive procedure? A definition to inform study design, evidence synthesis and research tracking. BMJ Open *9*, e028576.

38. U.S. Department of Health and Human Services (2019). Food and Drug Administration, Center for Drug Evaluation and Research, Center for Biologics Evaluation and Research. In Immunogenicity Testing of Therapeutic Protein Products — Developing and Validating Assays for Anti-Drug Antibody Detection, pp. 22–25, https://www.fda.gov/regulatory-information/search-fda-guidance-documents/immunogenicity-testing-therapeutic-protein-products-developing-and-validating-assays-anti-drug.

39. Mora, J., Palmer, R., Wagner, L., Wu, B., Partridge, M., Dakappagari, N., Meena, Sonderegger, I., Sonderegger, I., Smeraglia, J., et al. (2024). 2023 White Paper on Recent Issues in Bioanalysis: ISR for ADA Assays, the Rise of dPCR vs qPCR, International Reference Standards for Vaccine Assays, Anti-AAV TAb Post-Dose Assessment, NanoString Validation, ELISpot as Gold Standard (Part 3 - Recommendations on Gene Therapy, Cell Therapy, Vaccines Immunogenicity & Technologies; Biotherapeutics Immunogenicity & Risk Assessment; ADA/NAb Assay/Reporting Harmonization). Bioanalysis *16*, 77–119.

40. Loo, L., Harris, S., Milton, M., Lembke, W., Lembke, W., Berisha, F., Bertholet, S., Dessy, F., Dodge, R., Fang, X., et al. (2022). 2021 White Paper on Recent Issues in Bioanalysis: TAb/NAb, Viral Vector CDx, Shedding Assays; CRISPR/Cas9 & CAR-T Immunogenicity; PCR & Vaccine Assay Performance; ADA Assay Comparability & Cut Point Appropriateness (Part 3 - Recommendations on Gene Therapy, Cell Therapy, Vaccine Assays; Immunogenicity of Biotherapeutics and Novel Modalities; Integrated Summary of Immunogenicity Harmonization). Bioanalysis *14*, 737–793.

41. Myler, H., Pedras-Vasconcelos, J., Phillips, K., Hottenstein, C.S., Chamberlain, P., Devanaryan, V., Gleason, C., Goodman, J., Manning, M.S., Purushothama, S., et al. (2021). Anti-drug Antibody Validation Testing and Reporting Harmonization. AAPS J. *24*, 4.

42. Minh, B.Q., Schmidt, H.A., Chernomor, O., Schrempf, D., Woodhams, M.D., von Haeseler, A., and Lanfear, R. (2020). IQ-TREE 2: New Models and Efficient Methods for Phylogenetic Inference in the Genomic Era. Mol. Biol. Evol. *37*, 1530–1534.

43. Shi, Y.K.G., Soukhavong, D., Lamb, J., Meng, Q., Finley, T., Wang, T., Chen, W., Ma, W., Ye, Q., Liu, T., et al. (2026). lightgbm: Light Gradient Boosting Machine. R package version 46099. https://github.com/lightgbm-org/LightGBM.

OMTA, Volume *34*

Supplemental information

Seroprevalence of anti-AAV neutralizing antibodies in healthy and retinitis pigmentosa cohorts: A multi-province study in China

Jingxiao Du, Huixun Jia, Xuewei Xie, Xiaosa Li, Ting Zhang, Yidong Wu, Zishi Liu, Haoliang Zhang, Zhiyan Li, Weihua Gu, Xinkun Tao, Jieqiong Chen, Junran Sun, Tong Li, and Xiaodong Sun

SUPPLEMENTAL MATERIAL

Supplemental Figures

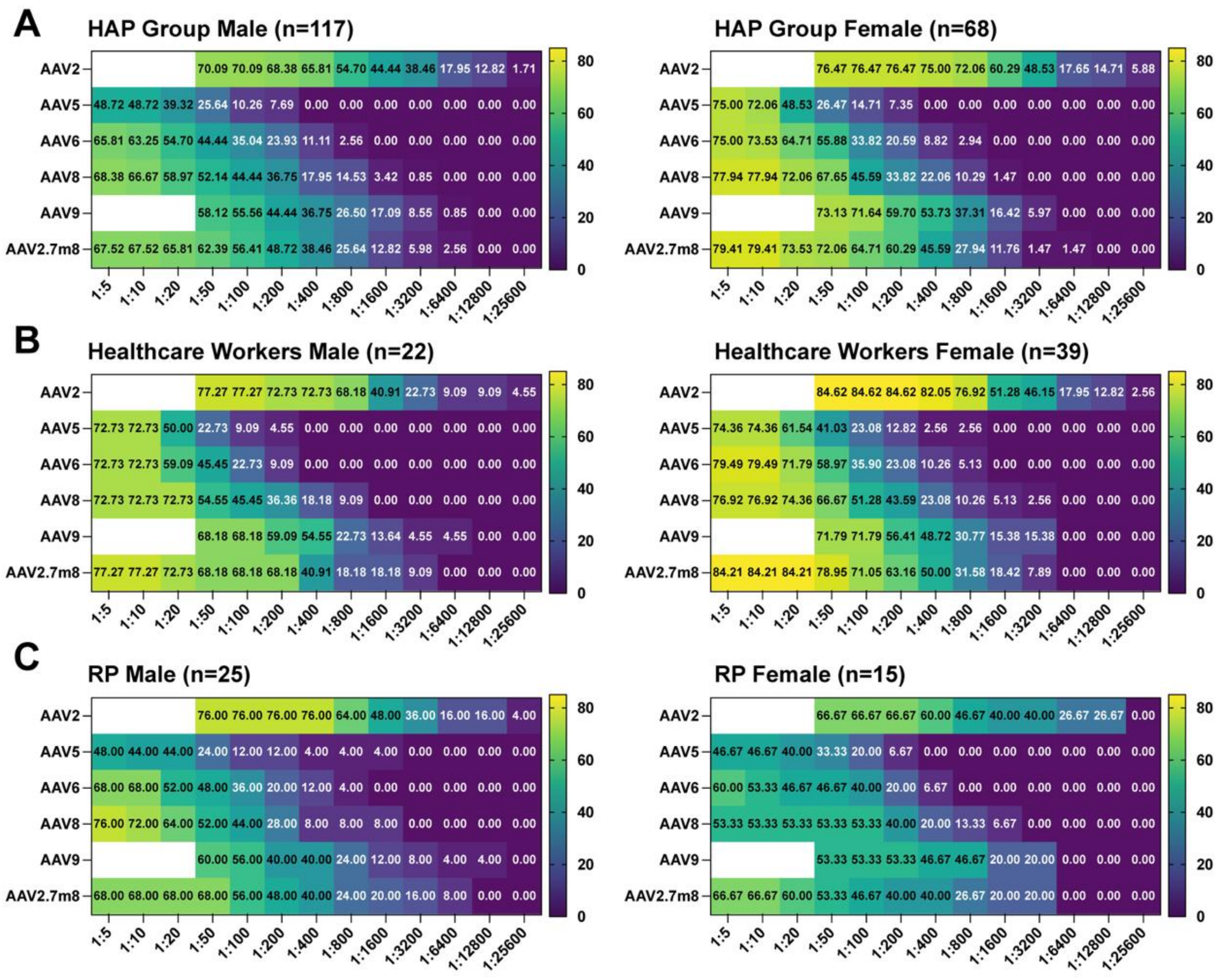

Figure S1. Gender-specific NAbs seroprevalence across different dilution gradients in the HAP, healthcare workers, and RP groups.

(A) Heatmap of anti-AAV NAbs seroprevalence by sex in HAP group for AAV2, AAV5, AAV6, AAV8, AAV9, and AAV2.7m8.

(B) Heatmap of anti-AAV NAbs seroprevalence by sex in healthcare workers for AAV2, AAV5, AAV6, AAV8, AAV9, and AAV2.7m8.

(C) Heatmap of anti-AAV NAbs seroprevalence by sex in RP group for AAV2, AAV5, AAV6, AAV8, AAV9, and AAV2.7m8.

The percentage of seropositive individuals is indicated by color intensity according to the scale, with numbers inside squares representing the exact proportion at each serotype and dilution level.

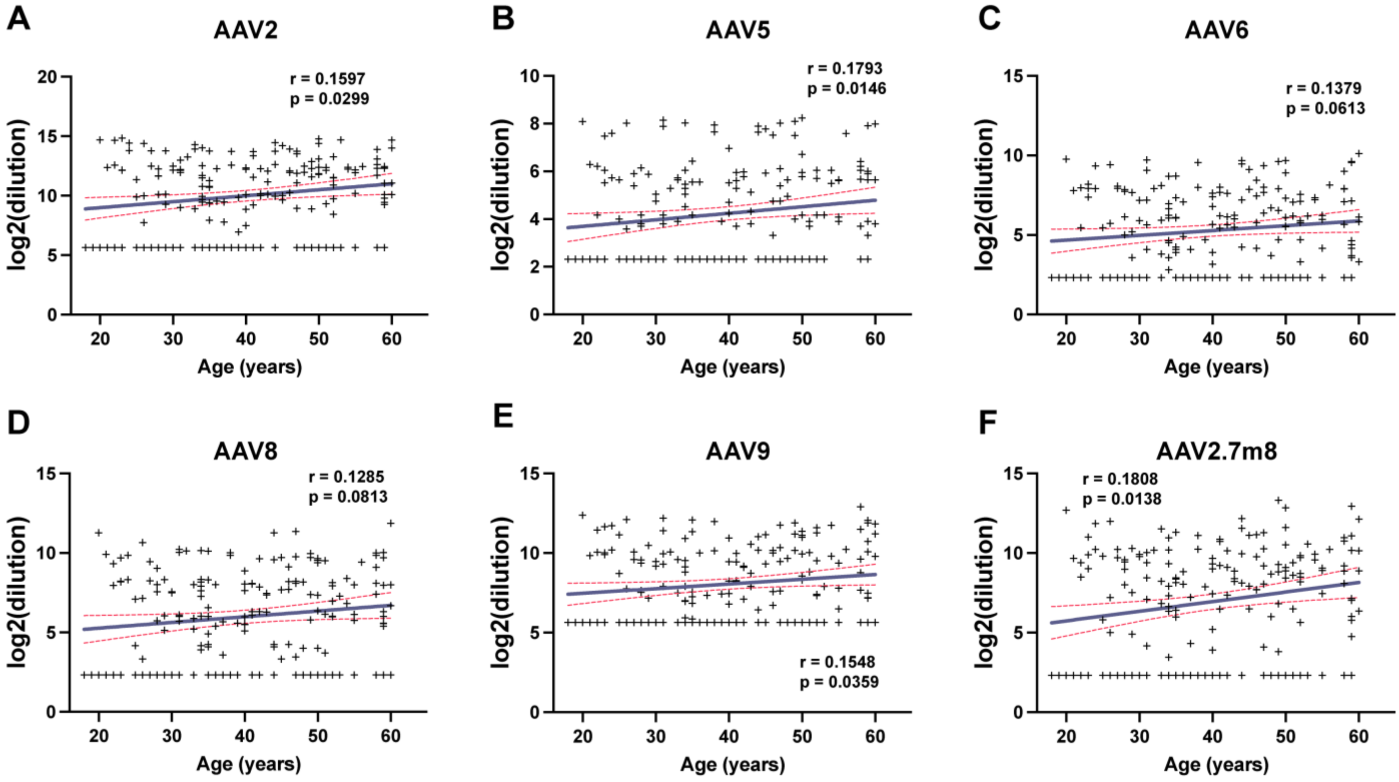

Figure S2. Correlations between anti-AAV NAb titers and age in the HAP group.

(A–F) Scatter plots illustrating the correlations between NAb titers against AAV2, AAV5, AAV6, AAV8, AAV9, and AAV2.7m8 and participant age. Blue solid line: fitted simple linear regression. Red dashed line: 95% confidence interval for the fitted regression. r, Spearman's correlation coefficient. Correlations were calculated using log2-transformed NAb titers (ID50).

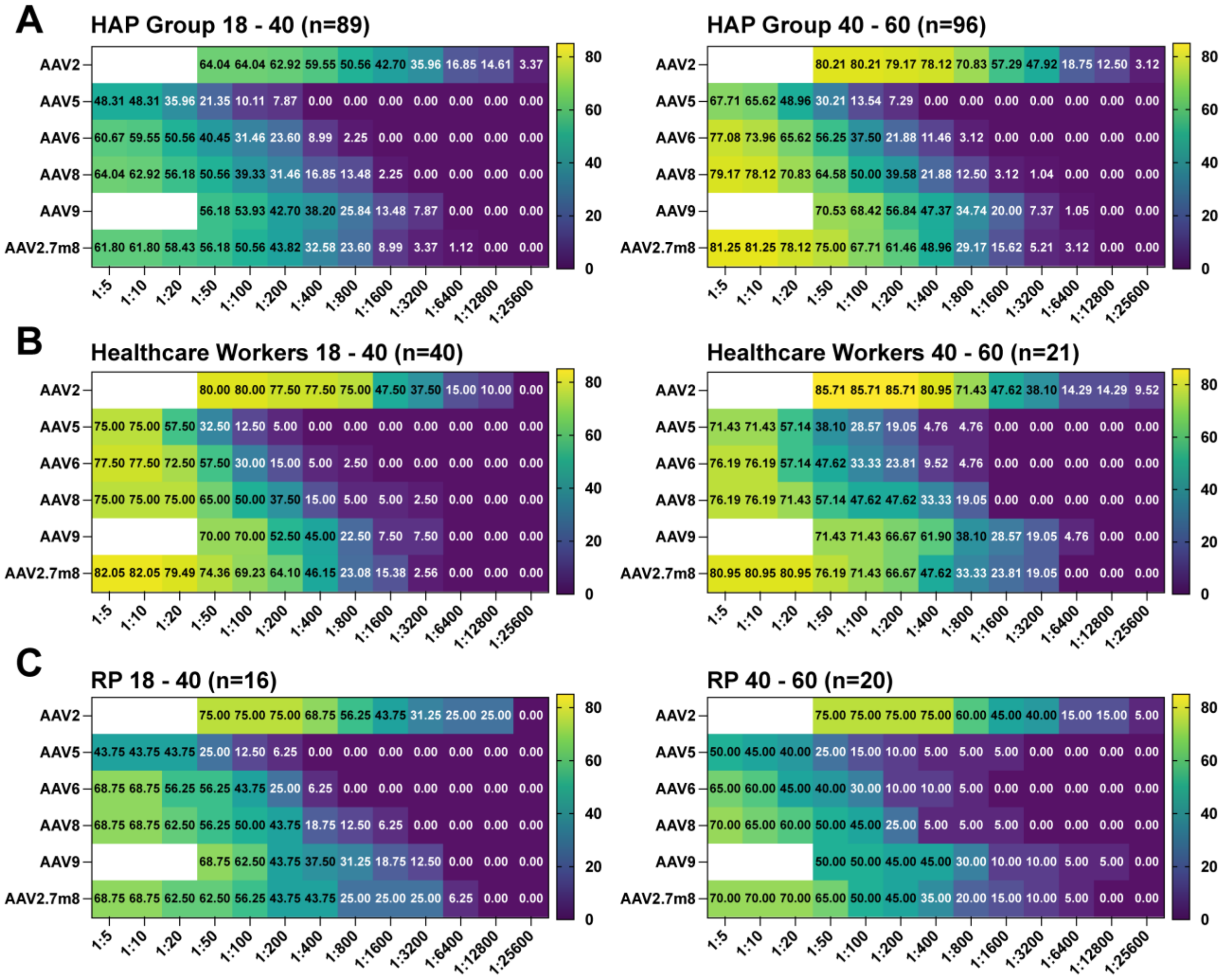

Figure S3. Age-specific anti-AAV NAb seroprevalence across dilution gradients in the HAP, healthcare worker, and RP groups.

(A) Heatmap of age-specific anti-AAV NAb seroprevalence in the HAP group for AAV2, AAV5, AAV6, AAV8, AAV9, and AAV2.7m8.

(B) Heatmap of age-specific anti-AAV NAb seroprevalence in healthcare workers for AAV2, AAV5, AAV6, AAV8, AAV9, and AAV2.7m8.

(C) Heatmap of age-specific anti-AAV NAb seroprevalence in the RP group for AAV2, AAV5, AAV6, AAV8, AAV9, and AAV2.7m8.

The percentage of seropositive individuals is indicated by color intensity according to the scale, with numbers inside squares representing the exact proportion at each serotype and dilution level.

Supplemental Tables

Table S1. NAbs characteristics by gender in adult controls, healthcare workers and RP patients

Group	Serotype	Gender	Median	95% CI	Minimum	Maximum	p-value
HAP	AAV2	Male	1:998	1:665 - 1:2903	1:50	1:26578	0.152
		Female	1:2612	1:1115 - 1:4548	1:50	1:29400	
	AAV5	Male	1:5	1:5 - 1:18	1:5	1:273	**0.011**
		Female	1:19	1:15 - 1:50	1:5	1:302	
	AAV6	Male	1:37	1:15 - 1:70	1:5	1:1109	0.388
		Female	1:58	1:25 - 1:73	1:5	1:843	
	AA8	Male	1:59	1:19 - 1:154	1:5	1:3724	0.356
		Female	1:78	1:63 - 1:181	1:5	1:2617	
	AAV9	Male	1:144	1:50 - 1:242	1:50	1:7654	**0.056**
		Female	1:452	1:185 - 1:773	1:50	1:4686	
	AAV2.7m8	Male	1:164	1:89 - 1:344	1:5	1:10240	0.231
		Female	1:342	1:174 - 1:534	1:5	1:7360	
Healthcare workers	AAV2	Male	1:1146	1:197 - 1:2914	1:50	1:28855	0.42
		Female	1:2402	1:915 - 1:3968	1:50	1:46936	
	AAV5	Male	1:26	1:5 - 1:50	1:5	1:232	0.255
		Female	1:50	1:13 - 1:65	1:5	1:1269	
	AAV6	Male	1:33	1:5 - 1:94	1:5	1:319	0.21
		Female	1:66	1:31 - 1:132	1:5	1:1069	
	AA8	Male	1:66	1:5 - 1:263	1:5	1:1383	0.57
		Female	1:127	1:47 - 1:269	1:5	1:3243	

	AAV9	Male	1:491	1:50 - 1:787	1:50	1:6409	0.698
		Female	1:298	1:123 - 1:792	1:50	1:4918	
	AAV2.7m8	Male	1:364	1:16 - 1:532	1:5	1:4141	0.639
		Female	1:327	1:145 - 1:675	1:5	1:5704	
RP	AAV2	Male	1:1246	1:685 - 1:3775	1:50	1:27487	0.756
		Female	1:773	1:50 - 1:13904	1:50	1:21360	
	AAV5	Male	1:5	1:5 - 1:31	1:5	1:2166	0.988
		Female	1:5	1:5 - 1:70	1:5	1:256	
	AAV6	Male	1:34	1:5 - 1:155	1:5	1:1150	0.679
		Female	1:17	1:5 - 1:152	1:5	1:593	
	AA8	Male	1:57	1:17 - 1:197	1:5	1:2536	0.932
		Female	1:156	1:5 - 1:331	1:5	1:2774	
	AAV9	Male	1:176	1:50 - 1:576	1:50	1:23381	0.581
		Female	1:320	1:50 - 1:1080	1:50	1:4916	
	AAV2.7m8	Male	1:156	1:5 - 1:472	1:5	1:9716	0.798
		Female	1:97	1:5 - 1:977	1:5	1:4219	

HAP, healthy adult participants; RP, retinitis pigmentosa patients; 95% CI, 95% confidence interval.

Table S2. NAbs characteristics by age groups in adult controls, healthcare workers and RP patients

Group	Serotype	Age	Median	95% CI	Minimum	Maximum	p-value
HAP	AAV2	18 to 40	1:813	1:221 - 1:2467	1:50	1:29400	**0.031**
		40 to 60	1:3001	1:1115 - 1:4350	1:50	1:28095	
	AAV5	18 to 40	1:5	1:5 - 1:18	1:5	1:284	**0.022**
		40 to 60	1:19	1:14 - 1:49	1:5	1:302	
	AAV6	18 to 40	1:23	1:5 - 1:60	1:5	1:874	**0.053**
		40 to 60	1:61	1:43 - 1:83	1:5	1:1109	
	AA8	18 to 40	1:51	1:15 - 1:136	1:5	1:2458	0.079
		40 to 60	1:92	1:63 - 1:206	1:5	1:3724	
	AAV9	18 to 40	1:144	1:50 - 1:242	1:50	1:5313	**0.062**
		40 to 60	1:308	1:175 - 1:694	1:50	1:7654	
	AAV2.7m8	18 to 40	1:114	1:11 - 1:284	1:5	1:6597	**0.011**
		40 to 60	1:376	1:207 - 1:534	1:5	1:10240	
Healthcare workers	AAV2	18 to 40	1:1275	1:956 - 1:3333	1:50	1:19825	0.813
		40 to 60	1:1547	1:797 - 1:4218	1:50	1:46936	
	AAV5	18 to 40	1:41	1:15 - 1:50	1:5	1:305	0.667
		40 to 60	1:50	1:5 - 1:162	1:5	1:1269	
	AAV6	18 to 40	1:63	1:31 - 1:82	1:5	1:1068	0.891
		40 to 60	1:36	1:13 - 1:179	1:5	1:1069	
	AA8	18 to 40	1:99	1:44 - 1:220	1:5	1:3243	0.449
		40 to 60	1:72	1:12 - 1:663	1:5	1:1383	
	AAV9	18 to 40	1:235	1:126 - 1:694	1:50	1:4918	0.199
		40 to 60	1:726	1:50 - 1:1631	1:50	1:6409	

	AAV2.7m8	18 to 40	1:265	1:129 - 1:532	1:5	1:3782	0.41
		40 to 60	1:391	1:59 - 1:1543	1:5	1:5704	
RP	AAV2	18 to 40	1:1067	1:50 - 1:14938	1:50	1:20755	0.885
		40 to 60	1:1093	1:411 - 1:4011	1:50	1:27487	
	AAV5	18 to 40	1:5	1:5 - 1:55	1:5	1:262	0.836
		40 to 60	1:7	1:5 - 1:38	1:5	1:2166	
	AAV6	18 to 40	1:57	1:5 - 1:218	1:5	1:683	0.486
		40 to 60	1:18	1:5 - 1:101	1:5	1:1150	
	AA8	18 to 40	1:92	1:5 - 1:282	1:5	1:2536	0.572
		40 to 60	1:54	1:5 - 1:197	1:5	1:2774	
	AAV9	18 to 40	1:187	1:50 - 1:923	1:50	1:4495	0.729
		40 to 60	1:95	1:50 - 1:873	1:50	1:23381	
	AAV2.7m8	18 to 40	1:154	1:5 - 1:3613	1:5	1:7759	0.809
		40 to 60	1:123	1:5 - 1:436	1:5	1:9716	

HAP, healthy adult participants; RP, retinitis pigmentosa patients; 95% CI, 95% confidence interval.

Table S3. Validation of Test Method for AAV2 Neutralizing Antibodies

**Method parameters**	**Validation results**
**Test item**	Anti-adeno-associated virus type 2 (AAV2) neutralizing antibody
**Analytical method**	Cell-based assay based on HEK293T
**Matrix**	Normal human serum (Chinese origin)
**Minimum required dilution**	1:50
**Applicable sample**	Human serum samples
**Detection instrument**	Microplate reader (Spectra Max L)
**Data acquisition software**	SoftMax Pro GxP V5.4.1
**Data processing software**	Microsoft Office Excel (Version 2013)
**Titer positive control (TPC)**	
TPC1	1:50 dilution, 2500 ng/mL (98.9% inhibition)
TPC2	1:250 dilution, 500 ng/mL (75.4% inhibition)
TPC3	1:1250 dilution, 100 ng/mL (24.2% inhibition)
TPC4	1:6250 dilution, 20.0 ng/mL (3.6% inhibition)
**Titer negative control**	Pooled serum showed baseline luciferase activity when mixed with AAV-containing cell culture media
**Intra-assay precision**	
TPC signal CV	2.7%~32.8%
NC signal CV	1.5%~14.9%
TPC titer (NT50) CV	5.1%~14.2%
MSR	1.160~1.535
**Inter-assay precision**	
TPC titer (NT50) CV	15.2%
MSR	1.428
**Sensitivity**	379 ng/mL
**Selectivity in matrix**	
Normal human serum sample	No interference detected
Lipaemic sample(300 mg/dL of triglycerides)	No interference detected
Haemolysed sample (2 % human whole blood)	No interference detected
**Drug tolerance**	2000 ng/mL positive control sample tolerated 1.00×10^{11} vg/mL virus
	1000 ng/mL positive control sample tolerated 1.00×10^{11} vg/mL virus

**Robustness meets acceptance criteria**	
Sample incubation time	1 hour ± 5 minutes
Cell incubation time	24 hours ± 2 hours
color development time	20 minutes ± 5 minutes
**Short-term stability**	
Room temperature	up to 24.5 hours
2-8°C	up to 72 hours
Freeze/thaw (-90 ℃~-60 ℃)	3 or 6 cycles
**Long-term stability**	
-25℃~-15℃	95 days
-90℃~-60℃	95 days
**Note**	Per USP 40 1106, WRIB white paper and European Bioanalysis Forum consensus: ADA considered stable ≥2 years at ≤-20°C in matrix

TPC, titer positive control; NC, negative control; CV, coeffcient of variation; MSR, minimum significant ratio; NT50, titers neutralizing 50% of transduction; ADA, anti-drug antibody

Table S4. Validation of Test Method for AAV5 Neutralizing Antibodies

**Method parameters**	**Validation results**
**Test item**	Anti-adeno-associated virus type 5 (AAV5) neutralizing antibody
**Analytical method**	Cell-based assay based on HEK293T
**Matrix**	Normal human serum (Chinese origin)
**Minimum required dilution**	1:5
**Applicable sample**	Human serum samples
**Detection instrument**	Microplate reader (Spectra Max L)
**Data acquisition software**	SoftMax Pro GxP V5.4.1
**Data processing software**	Microsoft Office Excel (Version 2013 or higher)
**Titer positive control (TPC)**	
TPC1	1:5 dilution, 800 ng/mL (98.5% inhibition)
TPC2	1:20 dilution, 200 ng/mL (73.4% inhibition)
TPC3	1:80 dilution, 50.0 ng/mL (30.4% inhibition)
TPC4	1:320 dilution, 12.5 ng/mL (7.8% inhibition)
**Titer negative control**	Pooled serum showed baseline luciferase activity when mixed with AAV-containing cell culture media
**Intra-assay precision**	
TPC signal CV	2.6%~24.4%
NC signal CV	3.1%~8.1%
TPC titer (NT50) CV	3.0%~12.2%
MSR	1.089~1.407
**Inter-assay precision**	
TPC titer (NT50) CV	9.4%
MSR	1.248
**Sensitivity**	138 ng/mL
**Selectivity in matrix**	
Normal human serum sample	No interference detected
Lipaemic sample(300 mg/dL of triglycerides)	No interference detected
Haemolysed sample (2 % human whole blood)	No interference detected
**Drug tolerance**	800 ng/mL positive control sample tolerated 1.00×10^{11} vg/mL virus
	200 ng/mL positive control sample tolerated 1.00×10^{11} vg/mL virus
**Robustness meets acceptance criteria**	

Sample incubation time	1 hour ± 5 minutes
Cell incubation time	24 hours ± 2 hours
color development time	NA
**Short-term stability**	
Room temperature	up to 24 hours
2-8°C	up to 73 hours
Freeze/thaw (-90 ℃~-60 ℃)	3 or 6 cycles
**Long-term stability**	
-25℃~-15℃	108 days
-90℃~-60℃	108 days
**Note**	Per USP 40 1106, WRIB white paper and European Bioanalysis Forum consensus: ADA considered stable ≥2 years at ≤-20°C in matrix

TPC, titer positive control; NC, negative control; CV, coeffcient of variation; MSR, minimum significant ratio; NT50, titers neutralizing 50% of transduction; ADA, anti-drug antibody

Table S5. Validation of Test Method for AAV6 Neutralizing Antibodies

Method parameters	Validation results
**Test item**	Anti-adeno-associated virus type 6 (AAV6) neutralizing antibody
**Analytical method**	Cell-based assay based on HEK293T
**Matrix**	Normal human serum (Chinese origin)
**Minimum required dilution**	1:5
**Applicable sample**	Human serum samples
**Detection instrument**	Microplate reader (Spectra Max L)
**Data acquisition software**	SoftMax Pro GxP 7.2
**Data processing software**	Microsoft Office Excel (Version 2016)
**Titer positive control (TPC)**	
TPC1	1:5 dilution, 1000 ng/mL (99.1% inhibition)
TPC2	1:20 dilution, 250 ng/mL (60.7% inhibition)
TPC3	1:80 dilution, 62.5 ng/mL (21.2% inhibition)
TPC4	1:320 dilution, 15.6 ng/mL (8.2% inhibition)
**Titer negative control**	Pooled serum showed baseline luciferase activity when mixed with AAV-containing cell culture media
**Intra-assay precision**	
TPC signal CV	0.7%~20.9%
NC signal CV	2.4%~5.1%
TPC titer (NT50) CV	2.5%~10.7%
MSR	1.073~1.381
**Inter-assay precision**	
TPC titer (NT50) CV	0.127
MSR	1.358
**Sensitivity**	225 ng/mL
**Selectivity in matrix**	
Normal human serum sample	No interference detected
Lipaemic sample(300 mg/dL of triglycerides)	No interference detected
Haemolysed sample (2 % human whole blood)	No interference detected
**Drug tolerance**	1000 ng/mL positive control sample tolerated 1.00×10^{11} vg/mL virus
	500 ng/mL positive control sample tolerated 1.00×10^{11} vg/mL virus

**Robustness meets acceptance criteria**	
Sample incubation time	1 hour ± 5 minutes
Cell incubation time	24 hours ± 2 hours
color development time	NA
**Short-term stability**	
Room temperature	up to 25 hours
2-8°C	up to 72 hours
Freeze/thaw (-90 ℃~-60 ℃)	3 or 6 cycles
**Long-term stability**	
-25℃~-15℃	NA
-90℃~-60℃	NA
**Note**	Per USP 40 1106, WRIB white paper and European Bioanalysis Forum consensus: ADA considered stable ≥2 years at ≤-20°C in matrix

TPC, titer positive control; NC, negative control; CV, coeffcient of variation; MSR, minimum significant ratio; NT50, titers neutralizing 50% of transduction; ADA, anti-drug antibody

Table S6. Validation of Test Method for AAV8 Neutralizing Antibodies

Method parameters	Validation results
**Test item**	Anti-adeno-associated virus type 8 (AAV8) neutralizing antibody
**Analytical method**	Cell-based assay based on HEK293T
**Matrix**	Normal human serum (Chinese origin)
**Minimum required dilution**	1:5
**Applicable sample**	Human serum samples
**Detection instrument**	Microplate reader (Spectra Max L)
**Data acquisition software**	SoftMax Pro GxP V5.4.1
**Data processing software**	Microsoft Office Excel (Version 2016)
**Titer positive control (TPC)**	
TPC1	1:5 dilution, 200 ng/mL (96.1% inhibition)
TPC2	1:20 dilution, 50.0 ng/mL (60.0% inhibition)
TPC3	1:80 dilution, 12.5 ng/mL (17.5% inhibition)
TPC4	1:320 dilution, 3.13 ng/mL (5.6% inhibition)
**Titer negative control**	Pooled serum showed baseline luciferase activity when mixed with AAV-containing cell culture media
**Intra-assay precision**	
TPC signal CV	2.0%~18.7%
NC signal CV	1.8%~6.7%
TPC titer (NT50) CV	4.2%~20.2%
MSR	1.127~1.937
**Inter-assay precision**	
TPC titer (NT50) CV	16.5%
MSR	1.525
**Sensitivity**	48.2 ng/mL
**Selectivity in matrix**	
Normal human serum sample	No interference detected
Lipaemic sample(300 mg/dL of triglycerides)	No interference detected
Haemolysed sample (2 % human whole blood)	No interference detected
**Drug tolerance**	200 ng/mL positive sample tolerated up to 1.00×10^{11} vg/mL drug
	100 ng/mL positive sample tolerated up to 1.00×10^{10} vg/mL drug

**Robustness meets acceptance criteria**	
Sample incubation time	1 hour ± 5 minutes
Cell incubation time	24 hours ± 2 hours
color development time	NA
**Short-term stability**	
Room temperature	up to 28 hours 7 minutes
2-8°C	up to 76 hours 8 minutes
Freeze/thaw (-90 ℃~-60 ℃)	3 or 6 cycles
**Long-term stability**	
-25℃~-15℃	112 days
-90℃~-60℃	112 days
**Note**	Per USP 40 1106, WRIB white paper and European Bioanalysis Forum consensus: ADA considered stable ≥2 years at ≤-20°C in matrix

TPC, titer positive control; NC, negative control; CV, coeffcient of variation; MSR, minimum significant ratio; NT50, titers neutralizing 50% of transduction; ADA, anti-drug antibody

Table S7. Validation of Test Method for AAV9 Neutralizing Antibodies

Method parameters	Validation results
**Test item**	Anti-adeno-associated virus type 9 (AAV9) neutralizing antibody
**Analytical method**	Cell-based assay based on HEK293T
**Matrix**	Normal human serum (Chinese origin)
**Minimum required dilution**	1:50
**Applicable sample**	Human serum samples
**Detection instrument**	Microplate reader (Spectra Max L)
**Data acquisition software**	SoftMax Pro GxP V5.4.1
**Data processing software**	Microsoft Office Excel (Version 2013 or higher)
**Titer positive control (TPC)**	
TPC1	1:50 dilution, 2000 ng/mL (98.5% inhibition)
TPC2	1:250 dilution, 400 ng/mL (76.1% inhibition)
TPC3	1:1250 dilution, 80.0 ng/mL (30.6% inhibition)
TPC4	1:6250 dilution, 16.0 ng/mL (4.6% inhibition)
**Titer negative control**	Pooled serum showed baseline luciferase activity when mixed with AAV-containing cell culture media
**Intra-assay precision**	
TPC signal CV	3.3%~21.6%
NC signal CV	4.0%~9.4%
TPC titer (NT50) CV	4.1%~17.0%
MSR	1.124~1.601
**Inter-assay precision**	
TPC titer (NT50) CV	12.9%
MSR	1.346
**Sensitivity**	279 ng/mL
**Selectivity in matrix**	
Normal human serum sample	No interference detected
Lipaemic sample(300 mg/dL of triglycerides)	No interference detected
Haemolysed sample (2 % human whole blood)	No interference detected
**Drug tolerance**	2000 ng/mL positive control sample tolerated 1.00×10^{11} vg/mL virus
	1000 ng/mL positive control sample tolerated 1.00×10^{11} vg/mL virus

**Robustness meets acceptance criteria**	
Sample incubation time	1 hour ± 5 minutes
Cell incubation time	22 hours ± 2 hours
color development time	NA
**Short-term stability**	
Room temperature	up to 26 hours
2-8°C	up to 74 hours
Freeze/thaw (-90 ℃~-60 ℃)	3 or 6 cycles
**Long-term stability**	
-25℃~-15℃	NA
-90℃~-60℃	NA
**Note**	Per USP 40 1106, WRIB white paper and European Bioanalysis Forum consensus: ADA considered stable ≥2 years at ≤-20°C in matrix

TPC, titer positive control; NC, negative control; CV, coeffcient of variation; MSR, minimum significant ratio; NT50, titers neutralizing 50% of transduction; ADA, anti-drug antibody

Table S8. Validation of Test Method for AAV2.7m8 Neutralizing Antibodies

**Method parameters**	**Validation results**
**Test item**	Anti-adeno-associated virus type 2 mutation (AAV2.7m8) neutralizing antibody
**Analytical method**	Cell-based assay based on HEK293T
**Matrix**	Normal human serum (Chinese origin)
**Minimum required dilution**	1:5
**Applicable sample**	Human serum samples
**Detection instrument**	Microplate reader (Spectra Max L)
**Data acquisition software**	SoftMax Pro GxP V5.4.1
**Data processing software**	Microsoft Office Excel (Version 2016)
**Titer positive control (TPC)**	
TPC1	1:5 dilution, 800 ng/mL (97.9% inhibition)
TPC2	1:10 dilution, 400 ng/mL (85.7% inhibition)
TPC3	1:20 dilution, 200 ng/mL (53.6% inhibition)
TPC4	1:40 dilution, 100 ng/mL (26.1% inhibition)
**Titer negative control**	Pooled serum showed baseline luciferase activity when mixed with AAV-containing cell culture media
**Intra-assay precision**	
TPC signal CV	3.5%~22.9%
NC signal CV	2.8%~7.5%
TPC titer (NT50) CV	4.8%~13.5%
MSR	1.148~1.481
**Inter-assay precision**	
TPC titer (NT50) CV	15.7%
MSR	1.451
**Sensitivity**	224 ng/mL
**Selectivity in matrix**	
Normal human serum sample	No interference detected
Lipaemic sample (300 mg/dL of triglycerides)	No interference detected
Haemolysed sample (2 % human whole blood)	No interference detected
**Drug tolerance**	800 ng/mL positive samples can tolerate up to 1.00×10^{10} gc/mL drug
	400 ng/mL positive samples can tolerate up to 1.00×10^{9} gc/mL drug

**Robustness meets acceptance criteria**	
Sample incubation time	1 hour ± 5 minutes
Cell incubation time	24 hours ± 2 hours
color development time	NA
**Short-term stability**	
Room temperature	up to 25.5 hours
2-8°C	up to 72.5 hours
Freeze/thaw (-90 ℃~-60 ℃)	3 or 6 cycles
**Long-term stability**	
-25℃~-15℃	NA
-90℃~-60℃	NA
**Note**	Per USP 40 1106, WRIB white paper and European Bioanalysis Forum consensus: ADA considered stable ≥2 years at ≤-20°C in matrix

TPC, titer positive control; NC, negative control; CV, coeffcient of variation; MSR, minimum significant ratio; NT50, titers neutralizing 50% of transduction; ADA, anti-drug antibody

Table S9. MOI and baseline luciferase activity in transduction inhibition-based NAb assays for each NAb serotype.

NAb serotype	AAV2	AAV5	AAV6	AAV8	AAV9	AAV2.7m8
AAV infection MOI (vg/cell)	1500	16000	16000	10000	12400	1000
Baseline signal (RLU, NC from method validation)	98699~ 234346	1126342~1704036	3508517~ 10143014	2588869~ 5055186	111575~ 265109	2392807~3824444

MOI, multiplicity of infection; vg, viral genomes; RLU, relative light units; NC, negative control; AAV, adeno-associated virus; NAbs, neutralizing antibodies.

Supplemental Methods

Pseudo-Code for the Entire Feature Selection and Model Training

Algorithm: Recursive Feature Elimination for LightGBM Regression

Input:

- X_train, y_train: Training dataset features and target
- X_test, y_test: Test dataset features and target
- n_features_to_select: Minimum number of features (default: 1)
- step: Number of features to remove per iteration (default: 1)
- cv: Number of cross-validation folds (default: 5)
- lgbm_params: LightGBM hyperparameters

Output:

- best_features: List of selected feature names
- results_df: Performance metrics for each iteration
- cv_tables: Cross-validation RMSE and R^2 tables
- shap_values: SHAP values for feature interpretation

Procedure:

1. Initialize:
   - num_features ← total number of features
   - selected_features ← boolean array (all True)
   - results ← empty list

2. Set LightGBM parameters:
   - objective: 'regression'
   - num_leaves: 31
   - learning_rate: 0.05
   - n_estimators: 100

- random_state: 42

3. For each iteration (from num_features down to 1, step by step):

a. Select current features:

X_train_selected ← X_train[:, selected_features]

X_test_selected ← X_test[:, selected_features]

b. Perform K-Fold Cross-Validation:

For each fold in cv:

- Split training data into train_fold and validation_fold
- Train LightGBM model on train_fold
- Predict on validation_fold
- Calculate RMSE and R^2 for validation_fold
- Store fold results

- Calculate mean_RMSE, std_RMSE, SE_RMSE across folds
- Calculate mean_R^2, std_R^2, SE_R^2 across folds

c. Train model on full training set:

- Fit LightGBM on X_train_selected
- Predict on X_test_selected
- Calculate test_RMSE and test_R^2

d. Store iteration results:

- n_features, CV metrics, test metrics, selected_features

e. Feature elimination:

- Extract feature_importances from trained model
  - Identify least important feature
  - Set selected_features[least_important_idx] ← False
  - Remove least important feature

4. Identify optimal feature subset:
   - best_idx ← argmin(CV_RMSE_mean) across all iterations
   - best_features ← features corresponding to best_idx
   - Note: All features evaluated down to n=1 to capture complete performance curve and identify global minimum

5. Train final model with optimal features:
   a. Model training:
      - Fit LightGBM on X_train[best_features]
      - Evaluate on X_test[best_features]
      - Calculate final RMSE, MAE, R^2

   b. Feature interpretation using SHAP:
      - Initialize TreeExplainer with trained model
      - Calculate SHAP values for X_train[best_features]
      - Generate SHAP summary plot to visualize:
        * Feature contribution magnitude
        * Feature value impact on predictions
        * Relative importance of selected features

6. Return:
   - best_features (optimal feature subset)
   - results_df (performance metrics across all iterations)

- cv_rmse_table, cv_r2_table (detailed cross-validation results)
- shap_values (feature contribution analysis)
